# Copper overload drives hepatic stellate cell activation via cuproptosis to exacerbate acute liver injury in murine malaria

**DOI:** 10.1186/s13071-026-07476-0

**Published:** 2026-06-08

**Authors:** Xiumei Mo, Qian Chen, Rui Zhong, Yannan Lin, Zhiqi Liu, Xiaoran Zhang, Xinpeng Hou, Haofeng Lin, Min Zhang, Wenbin Liu, Jiajing He, Zhiyu Chen, Minqiu Ye, Zujun Deng, Xiaobao Jin, Zhenlong Liu, Jianping Song, Hui Yin, Bo Huang

**Affiliations:** 1 Guangdong Provincial Key Laboratory of Pharmaceutical Bioactive Substances, Guangdong Pharmaceutical University, Guangzhou, 510006 People’s Republic of China; 2 Department of Medical Laboratory, Shenzhen Traditional Chinese Medicine Hospital, Shenzhen, 518033 China; 3 Department of Medical Laboratory, The Second Affiliated Hospital of Guangdong Medical University, Zhanjiang, 514021 People’s Republic of China; 4 Laboratory Animal Center, Guangdong Pharmaceutical University, Guangzhou, 510006 People’s Republic of China; 5 Department of Critical Care Medicine, Foshan Sanshui District People’s Hospital, Foshan, 528199 China; 6 School of Basic Medical Science, Guangdong Pharmaceutical University, Guangzhou, 510006 People’s Republic of China; 7 Division of Experimental Medicine, Department of Medicine, McGill University, Montreal, QC Canada; 8 Artemisinin Research Center, Guangzhou University of Chinese Medicine, Guangzhou, 510405 People’s Republic of China

**Keywords:** *Plasmodium berghei* ANKA, Acute liver injury, Cuproptosis, Hepatic stellate cell, Copper homeostasis, Fibrosis

## Abstract

**Background:**

Acute liver injury, a critical complication of severe malaria, was characterized by hepatocyte death and hepatic stellate cell (HSC) activation. While HSC activation was a well-established driver of chronic liver fibrosis, its specific role and the triggers initiating it during the acute phase of malarial liver injury remain poorly defined. Notably, systemic copper accumulation occurred during *Plasmodium* infection and can induce cuproptosis, a novel form of copper-dependent mitochondrial cell death. Given the liver’s central role in copper metabolism and storage, coupled with the known sensitivity of HSC to copper perturbations, we hypothesized that malaria-induced hepatic copper overload may trigger cuproptosis, which subsequently acted as a key signal promoting HSC activation, thereby exacerbating acute liver injury.

**Methods:**

Using a *P. berghei* ANKA (*Pb*)-infected C57BL/6 mouse model, we modulated copper homeostasis in vivo with the copper ionophore disulfiram (DSF) and the copper chelator tetrathiomolybdate (TTM). *Parasitemia* and liver parasite burden were quantified to assess infection severity. Liver injury was systematically evaluated through histopathology, serum alanine transaminase/aspartate aminotransferase (ALT/AST) level, apoptosis detection (TUNEL), and fibrosis assessment (Sirius Red staining). Systemic inflammatory responses were assessed by measuring serum levels of tumor necrosis factor-alpha (TNF-α), interferon-gamma (IFN-γ), and interleukin (IL)-10 using Enzyme-Linked ImmunoSorbent Assay (ELISA) kits. Hepatic copper levels were measured by rubeanic acid staining and inductively coupled plasma mass spectrometry (ICP-MS). To elucidate the underlying mechanisms, we analyzed the expression of key cuproptosis markers (FDX1, DLAT, and DLST) and hepatic stellate cell (HSC) activation markers (alpha smooth muscle actin (α-SMA) and Collagen I) using immunohistochemistry and immunofluorescence. Furthermore, the mRNA levels of profibrotic/antifibrotic mediators in liver tissue were measured by quantitative polymerase chain reaction (qPCR) assay. Finally, to validate the mechanistic link between copper dysregulation and HSC activation, we conducted in vitro studies using *Pb*-infected red blood cells (iRBCs)-stimulated HSC cell line (HSCT6) treated with DSF-CuCl_2_ or TTM-CuCl_2_ complexes.

**Results:**

*Pb* infection triggered progressive hepatic copper accumulation and concomitant upregulation of key cuproptosis markers (FDX1, DLAT, and DLST), which paralleled worsening liver histopathology, enhanced HSC activation (α-SMA/Collagen I↑), and increased collagen deposition (fibrosis). Critically, pharmacological elevation of copper levels using DSF exacerbated this pathological cascade: it amplified copper overload, further increased key cuproptosis marker expression (FDX1/DLAT/DLST↑), elevated parasitemia and liver parasite burden, and intensified HSC activation (α-SMA/Collagen I↑), fibrosis, and liver pathology. Strikingly, DSF treatment significantly increased the proportion of activated (α-SMA^+^) HSCs co-expressing FDX1, DLAT, or DLST, and shifted the cytokine balance toward profibrosis (TGF-β/PDGF↑) while suppressing antifibrotic mediators (IFN-γ/HGF↓). In parallel, DSF treatment increased serum TNF-α and IFN-γ levels while decreasing IL-10. Conversely, copper chelation with TTM effectively reversed these effects: it reduced hepatic copper, suppressed key cuproptosis marker expression and the prevalence of co-labeled HSC, alleviated HSC activation and fibrosis, and consequently improved liver function. This central mechanistic link between copper dysregulation and HSC activation was directly validated in vitro: in iRBC-stimulated HSCT6, DSF-CuCl_2_ complexes potentiated the upregulation of cuproptosis markers (FDX1, DLAT, and DLST), HSC activation markers (α-SMA and Collagen I), and profibrotic cytokines (TGF-β and PDGF) while inhibiting antifibrotic cytokines (IFN-γ and HGF), whereas TTM-CuCl_2_ treatment consistently attenuated these iRBC-induced changes.

**Conclusions:**

Our findings demonstrate that malaria-induced hepatic copper overload triggers cuproptosis, which in turn directly activates HSC, thereby exacerbating acute liver injury. Pharmacological intervention with the copper chelator TTM effectively suppressed this pathogenic cascade and mitigated liver damage. Consequently, targeting copper-induced cuproptosis represents a novel therapeutic strategy for combating severe malaria-associated acute liver injury.

**Graphical Abstract:**

**Figure:**
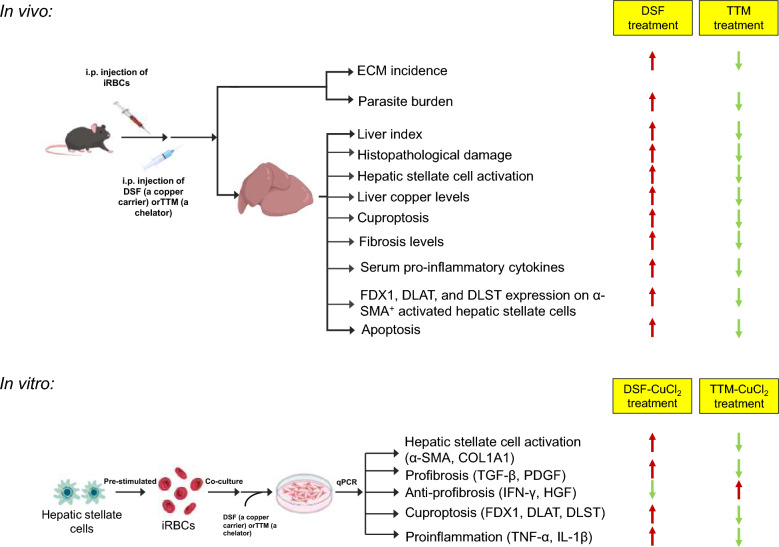

**Supplementary Information:**

The online version contains supplementary material available at 10.1186/s13071-026-07476-0.

## Background

Malaria, a life-threatening parasitic disease caused by *Plasmodium* spp. and transmitted by Anopheles mosquitoes, remained a catastrophic global health burden, with an estimated 263 million clinical cases and 597,000 deaths in 2023 [1]. The parasite’s complex life cycle dictates disease heterogeneity: following asymptomatic hepatocyte invasion and liver-stage development, the symptomatic blood stage, characterized by cyclical erythrocyte rupture, drove manifestations ranging from uncomplicated febrile illness to severe complications including acute liver injury [2]. This hepatic pathology, affecting up to 50% of severe malaria cases and presenting as hepatomegaly, jaundice, transaminase elevation, and centrilobular necrosis, significantly contributed to mortality by potentially precipitating multiorgan failure [3]. While hepatic sequestration of infected red blood cells (iRBCs), triggering endothelial activation, heme overload, and dysregulated inflammation, was a recognized driver of malarial liver injury, the precise molecular mechanisms remain poorly defined [4].

Liver homeostasis critically depended on the crosstalk between parenchymal hepatocytes and nonparenchymal cells (NPCs), encompassing liver sinusoidal endothelial cells, Kupffer cells, intrahepatic lymphocytes, and hepatic stellate cells (HSCs) [5]. NPCs served as primary targets for hepatotoxins and key mediators of physiological responses to endocrine and immune signals [6]. While HSCs were well-established master regulators of fibrogenesis in chronic liver diseases, emerging evidence highlights their significant, yet mechanistically underexplored, contribution to acute liver injury. In such contexts, early HSC activation can exacerbate inflammation and microvascular dysfunction through cytokine release (e.g., transforming growth factor beta (TGF-β) and interleukin (IL)-6) and extracellular matrix remodeling—phenomena documented in experimental models [7, 8]. Crucially, the specific molecular triggers and signaling pathways driving HSC activation during malaria-induced acute liver injury remained largely unknown.

Copper, an essential micronutrient serving as a catalytic cofactor in vital cellular processes such as mitochondrial respiration and antioxidant defense, was maintained at low intracellular concentrations due to its cytotoxic potential upon dysregulation [9]. This necessitated strict control over its uptake, distribution, and elimination [10]. The liver, with a copper concentration per gram of tissue higher than the brain, heart, and kidneys, stored the majority of the body’s copper and was thus especially susceptible to disruptions in copper homeostasis [11]. Such copper dysregulation triggered pathological cascades: intracellular copper overload induces cuproptosis—a recently identified copper-dependent cell death pathway characterized by mitochondrial copper accumulation and aggregation of lipoylated tricarboxylic acid (TCA) cycle proteins (e.g., DLAT and PDHB), leading to iron–sulfur cluster protein inactivation and a ferroptosis-like outcome [12]. Critically, copper overload also directly promoted HSC activation independently of cuproptosis [13]. In vivo evidence demonstrated copper-induced HSC proliferation, alpha smooth muscle actin (α-SMA) expression, and collagen synthesis via MAPK/ERK and JNK signaling, a profibrotic response conserved across liver injury models [14–16]. Importantly, multiple studies, including our recent data, revealed significant copper accumulation in sera and extrahepatic tissues (lung and brain) of malaria-infected hosts [17, 18]. This accumulation correlated with cuproptosis-associated tissue damage and enhanced *Plasmodium* proliferation in vitro [19]. Given the liver’s high physiological copper burden and central role in copper storage, coupled with the established paradigm of copper-driven HSC activation, we hypothesized that malaria-induced hepatic copper overload may precipitate cuproptosis in hepatocytes and/or sinusoidal endothelial cells. This, in turn, synergistically may exacerbate HSC activation, ultimately amplifying acute liver injury. To test this hypothesis and elucidate the role of cuproptosis in malaria-associated hepatic pathology, we utilized a *Pb*-infected C57BL/6 mouse model. We investigated the temporal expression patterns of key cuproptosis regulators and HSC activation markers following pharmacological modulation of copper homeostasis using the ionophore disulfiram (DSF) and the chelator tetrathiomolybdate (TTM), specifically examining the mechanistic link between HSC activation and cuproptosis.

## Methods

### Animal, parasite, and ethical approval

Female C57BL/6 mice (6 weeks old, weighing 18–20 g) sourced from the Guangdong Provincial Laboratory Animal Center were acclimatized and maintained under specific pathogen-free (SPF) conditions at the Guangdong Pharmaceutical University Laboratory Animal Center. Mice were housed in groups of five per cage within ventilated racks in a temperature-controlled room (25 °C) on a 12 h light/dark cycle, with free access to food and water. *Pb* parasites, obtained from Professor Jianping Song (Guangzhou University of Chinese Medicine), were cryopreserved as stabilates of infected red blood cells (iRBCs) in liquid nitrogen and subsequently used for infections. Cryopreserved parasites were thawed and injected intraperitoneally (i.p.) into donor mice; recipient mice were then infected by i.p. injection of an inoculum containing 1.0 × 10^5^ iRBCs in 200 µl phosphage buffer saline (PBS). All experimental procedures were approved by the Institutional Animal Care and Use Committee of Guangdong Pharmaceutical University (approval no. GDPULAC2023295), with all procedures conducted in strict adherence to the replacement, reduction, and refinement (3R) principles of animal welfare.

### Experimental design and animal handling protocols

A total of 96 female C57BL/6 mice were randomly allocated into 6 groups (*n* = 8 per group unless stated): the naïve group received daily i.p. injections of 100 μl 0.9% NaCl; the DSF group received daily i.p. injections of 50 mg/kg disulfiram (DSF, no. 86720, Sigma-Aldrich) in 100 μl NaCl; the TTM group received daily i.p. injections of 30 mg/kg tetrathiomolybdate (TTM, no. 323446, Sigma-Aldrich) in 100 μl NaCl; the *Pb* group (*n* = 24) was infected via i.p. inoculation with 1.0 × 10^5^ *Pb*-iRBCs and subsequently received daily i.p. injections of 100 μl NaCl starting the following day; the *Pb* + DSF group (*n* = 24) received *Pb* infection followed by daily i.p. DSF (50 mg/kg in 100 μl NaCl); and the *Pb* + TTM group (*n* = 24) received *Pb* infection followed by daily i.p. TTM (30 mg/kg in 100 μl NaCl). Throughout the study, mice were monitored daily for weight loss, survival time, blood parasitemia, and experimental cerebral malaria (ECM) neurological signs (including hemiparesis, ataxia, absent reflexes, convulsions, and coma). Body weights were recorded regularly. Starting on day 3 post-infection (3 dpi), parasitemia was assessed daily by microscopic examination of Giemsa-stained thin blood smears prepared from tail vein blood, calculated as (parasitized RBCs/total RBCs) × 100%. ECM diagnosis required the presence of neurological symptoms accompanied by mortality within 24 h of symptom onset (typically 6–11 dpi), whereas mice evading neurological symptoms often succumbed later (12–20 dpi) to hyperparasitemia-induced systemic pathology (e.g., severe anemia and multiorgan failure). All animals were assessed daily for distress signs, and time of death was promptly documented.

### Quantification of hepatic somatic index

On 8 and 15 dpi, mice were humanely euthanized via cervical dislocation under deep anesthesia and immediately weighed. The livers were then carefully excised intact, rinsed in ice-cold saline to remove blood, and gently blotted dry. Wet liver weights were measured promptly using a precision electronic balance to prevent desiccation. The hepatic somatic index, commonly referred to as the liver index (%), was calculated as liver weight (g)/body weight (g) × 100, a standard and established methodology for assessing hepatomegaly in preclinical models [20]. This index serves as a macroscopic marker of liver injury, with an elevated value reflecting liver enlargement resulting from inflammation, edema, or cellular hypertrophy [21, 22].

### Assessment of hepatic histopathology and systemic inflammatory response

On days 8 and 15 post-*Pb* infection, mice (*n* = 4–5 per group per timepoint) were anesthetized, and blood was collected for serum isolation. The mice were then euthanized by cervical dislocation, and liver tissues were immediately harvested. Serum alanine aminotransferase (ALT) and aspartate aminotransferase (AST) activities were measured using an automatic biochemistry analyzer with commercial assay kits (no. S03030 and S03040, Rayto), in accordance with the manufacturer’s instructions. Systemic inflammatory responses were assessed by measuring serum levels of tumor necrosis factor-alpha (TNF-α), interferon-gamma (IFN-γ), and interleukin-10 (IL-10) using Enzyme-Linked ImmunoSorbent Assay (ELISA) kits (Cusabio, China; TNF-α: no. CSB-E04741m, IFN-γ: no. CSB-E04578m-IS, IL-10: no. CSB-E04594m-IS) according to the manufacturer’s protocols. To investigate the effects of DSF and TTM on liver histopathology, we performed hematoxylin and eosin (H&E) staining following the protocol described previously [23]. Briefly, liver tissues were fixed in 4% neutral buffered paraformaldehyde (pH 7.4) for 48 h, dehydrated through a graded ethanol series, cleared in xylene, and embedded in paraffin. Serial sections (5 μm) were cut on a rotary microtome, stained with H&E, and examined under an Olympus BX53 light microscope at 200× magnification. For each animal (*n* = 4–5 per group), more than 20 non-overlapping fields were randomly selected, and the number of inflammatory foci was counted in a double-blind manner by two independent investigators. The liver histopathological score was expressed as the number of inflammatory foci per field [23].

### Sirius Red staining for collagen deposition in liver tissue

Collagen deposition in liver tissue was assessed using Sirius Red staining. Freshly harvested liver samples from mice (*n* = 4–5 per group per timepoint) were fixed in 10% neutral buffered formalin for 48 h at room temperature and paraffin-embedded. Serial 5-μm sections were dewaxed in xylene (2 × 10 min) and rehydrated through graded ethanol (100%, 95%, 80%, and 70%). Sections were incubated in Sirius Red Stain Solution A (no. G1078-1, Servicebio) at 65 °C for 30 min. Subsequently, they were rinsed under running tap water until the yellow color faded from the tissue. After gently blotting off excess water, the sections were immersed in Stain Solution B (no. G1078-2, Servicebio) for 2 min. Following another gentle rinse and blotting, they were transferred to Stain Solution C (no. G1078-3, Servicebio) for a 30-min immersion. Thereafter, the sections were subjected to a quick water rinse (1–2 s) and sequentially dehydrated through three baths of absolute ethanol, with 3 dips in each. Finally, the sections were cleared in fresh xylene for 5 min and mounted with neutral balsam. Collagen distribution was assessed under a bright-field microscope (Olympus BX53, 200×), showing collagen fibers in red, while muscle fibers and other tissue components appeared yellow. Collagen deposition was quantified using ImageJ/FIJI software by measuring the percentage of Sirius-Red-positive area per field. For each liver section, ≥ 20 non-overlapping fields were randomly captured at 200× magnification, encompassing portal tracts, centrilobular regions, and fibrotic septa within the hepatic parenchyma. To ensure anatomical consistency across experimental groups, all images were obtained from the same liver lobe, as interlobular heterogeneity in fibrosis distribution has been well documented.

### Detection of copper deposition by erythrocuprein histochemical staining and inductively coupled plasma mass spectrometry (ICP-MS) assay

On 8 and 15 dpi, dewaxed and rehydrated paraffin sections of harvested liver tissue were immersed in freshly prepared rubeanic acid copper (RAC) staining solution (0.1% Rubeanic acid ethanol solution (no. 379387, Sigma) mixed with 10% sodium acetate solution (no. 10018818, China National Pharmaceutical Group) at a 1:20 volume ratio) and stained at 37 °C in the dark for 2 h. Subsequently, sections underwent sequential dehydration through graded ethanol (100%, 95%, and 80%) and distilled water washes, followed by nuclear counterstaining with nuclear fast red (no. G1035, China National Pharmaceutical Group) for 3 min, final dehydration (100% ethanol), xylene clearing, and mounting with neutral resin. Copper deposits (appearing as dark greenish-black precipitates) and nuclei (staining red) were examined under an Olympus BX53 microscope at 200× magnification, with quantitative analysis performed using Image-Pro Plus 6.0 software (Media Cybernetics, Rockville, MD, USA) on ≥ 20 randomly selected non-overlapping fields per sample to determine integrated optical density per unit area (IOD/area) according to established protocols. Additionally, total copper concentration in liver tissue was determined by inductively coupled plasma mass spectrometry (ICP-MS; Agilent 7500cx; Agilent Technologies, Santa Clara, CA, USA) following immediate tissue digestion with MOS-grade HNO_3_, subsequent digestion with a 3:1 (v/v) mixture of concentrated HNO_3_ and MOS-grade HClO_4_, and dissolution in 4% HNO_3_, with results expressed as micrograms of copper per gram of wet liver weight.

### Immunohistochemical analysis of hepatic FDX1, DLAT, DLST, α-SMA, and collagen I expressions

Following dewaxing and hydration, liver tissue sections underwent sequential processing comprising antigen retrieval in sodium citrate buffer (pH 6.0), quenching of endogenous peroxidase activity with 3% H_2_O_2_ at 37 °C for 25 min, and blocking of nonspecific binding sites using goat serum. Sections were subsequently incubated overnight at 4 °C with the following primary antibodies diluted in blocking buffer: rabbit anti-FDX1 (1:200, no. 12592-1-AP, Proteintech), rabbit anti-DLAT (1:500, no. GB113649, Servicebio), rabbit anti-DLST (1:1000, no. GB114020, Servicebio), rabbit anti-α-SMA (1:200, no. 14395-1-AP, Proteintech), and rabbit anti-collagen I (1:200, no. GB11022-3-100, Servicebio). After thorough washing with PBS, sections were incubated with horseradish peroxidase (HRP)-conjugated goat anti-rabbit immunoglobulin (Ig)G secondary antibody (1:200, no. S0001, Affinity) at 37 °C for 60 min. Immunoreactivity was visualized using 3,3’-diaminobenzidine (DAB) substrate, followed by counterstaining with hematoxylin. Finally, sections were dehydrated through a graded ethanol series, cleared in xylene, and mounted with neutral resin. Positive immunostaining for FDX1, DLAT, DLST, α-SMA, and collagen I was quantified as the integrated optical density per unit area (IOD/area) using image analysis software [24].

### Immunofluorescence double staining for α-SMA^+^-FDX1^+^, α-SMA^+^-DLAT^+^, and α-SMA^+^-DLST^+^ activated hepatic stellate cells

Liver tissue sections were dewaxed in xylene (two changes, 15 min each) and rehydrated through a graded ethanol series (100% twice, 95%, 80%, 70%; 5 min each) followed by three 5-min ddH_2_O washes. Antigen retrieval was performed by heating sections in sodium citrate buffer (pH 6.0) using a microwave oven (medium–high power for 8 min to preheat the buffer, followed by 7 min at low power after placing sections). Sections were then cooled to room temperature naturally and washed three times with PBS (5 min each). Nonspecific binding sites were blocked with goat serum for 30 min at 37 °C. After removing the blocking serum, sections were incubated overnight at 4 °C with primary antibody cocktails diluted in PBS: for α-SMA^+^/FDX1^+^ cells: mouse anti-α-SMA (1:100, no.GB13044, Servicebio) and rabbit anti-FDX1 (1:200, no.12592-1-AP, Proteintech); for α-SMA^+^/DLAT^+^ cells: mouse anti-α-SMA (1:100) and rabbit anti-DLAT (1:500, no.GB113649, Servicebio); for α-SMA^+^/DLST^+^ cells: mouse anti-α-SMA (1:100) and rabbit anti-DLST (1:1000, no.GB114020, Servicebio). The following day, sections were rewarmed at 37 °C for 40 min and washed three times with PBS (8 min each). Subsequently, sections were incubated for 60 min at 37 °C with a mixture of secondary antibodies diluted in PBS: CoraLite 488-conjugated Goat Anti-Mouse IgG (H + L) (1:1000) and CoraLite 594-conjugated Goat Anti-Rabbit IgG (H + L) (1:200). After four 8-min PBS washes, sections were mounted using an anti-fade mounting medium containing DAPI. Fluorescent images were acquired using the EVOS M5000 Imaging System at 200× magnification.

### TUNEL staining for hepatic apoptotic changes

Apoptotic changes in the livers of *Pb*-infected mice treated with DSF or TTM were assessed using TUNEL staining. Paraffin-embedded liver sections (*n* = 4 ~ 5 per group per timepoint; three non-contiguous sections per animal) were deparaffinized in xylene and rehydrated through a graded ethanol series. Sections were then digested with 20 μg/ml proteinase K at 37 °C for 30 min. After rinsing in distilled water, sections were incubated with the TUNEL reaction mixture (CY5-biotinylated dUTP in TdT buffer; TUNEL Apoptosis Detection Kit CY5, no. G1502-50T, Servicebio) at 37 °C for 60 min. Nuclei were counterstained with 1 μg/ml DAPI at 37 °C for 10 min. Sections were coverslipped using anti-fade mounting medium (Beyotime) and visualized under a Leica DM 2500B fluorescence microscope equipped with appropriate filters. Fluorescent images (×200 magnification) captured TUNEL-positive apoptotic nuclei (CY5 channel, pseudo-colored red) and total nuclei (DAPI channel, blue). The apoptotic index was quantified as the mean number of TUNEL-positive cells per microscopic field, determined by counting > 20 randomly selected fields per section in a double-blind analysis.

### In vitro co-culture experiment of iRBC-stimulated HSCT6 cells with DSF-CuCl_2_ or TTM-CuCl_2_

The immortalized rat HSC line (HSCT6) was purchased from Chinese Academy of Sciences (no. 5301RAT-KCB07003YJ). HSCT6 cells were cultured in Dulbecco’s modified Eagle’s medium (DMEM) supplemented with 10% fetal bovine serum (FBS; no. A2720801, Gibco^™^) and 1% penicillin/streptomycin at 5% CO_2_ and 37 °C. When the cells attained a density of 10^5^ cells/mL, the HSCT6 cells were plated in 6-well plates (10^4^ cells/well), and then allowed to adhere for 6 h, then stimulated with 1 × 10^5^ iRBCs/well. After 24 h priming, cells were treated with: (1) 10 μM CuCl_2_ (vehicle control), (2) 10 μM CuCl_2_ + 20 nM DSF, or (3) 10 μM CuCl_2_ + 20 nM TTM for 24/48 h. Post-treatment, cells were washed thrice with ice-cold PBS (pH 7.4), lysed with TRIzol^™^ (no. 9109, TaKaRa Bio), and stored at −80 °C until RNA extraction.

### Quantitative assessment of parasitic burden in hepatic tissue and cytokines in hepatic tissue or HSCT6 cells by qPCR

Liver tissue samples or HSCT6 cells from each experimental group were homogenized in TRIzol reagent for total RNA (1000 ng) extraction, followed by cDNA synthesis using the PrimeScript^™^ First Strand cDNA Synthesis Kit (no. 6210B, TaKaRa Bio) according to the manufacturer’s protocol. Gene-specific primers (e.g., for TGF-β, PDGF, IFN-γ, HGF, α-SMA, COL1A1, TNF-α, IL-1β, FDX1, DLAT, DLST, and *P. berghei* ANKA 18S rRNA) were commercially designed and synthesized by Sangon Biotech (Shanghai, China) Co., Ltd. All primer sequences are listed in Table S1. Quantitative real-time polymerase chain reaction (PCR) was performed using SYBR Green Premix Ex Taq^™^ (no. 6110B, TaKaRa Bio, Japan) on a CFX96 system (Bio-Rad), with GAPDH serving as the endogenous control. The thermal cycling conditions consisted of an initial denaturation at 95 °C for 1 min, followed by 40 cycles of 95 °C for 5 s and 60 °C for 20 s, with each sample run in triplicate. Relative gene expression was analyzed using the 2^−ΔΔCt^ method.

### Statistical analysis

The experimental data were analyzed using GraphPad Prism 8.0 software. Quantitative data were expressed as mean ± standard deviation (SD). Statistical comparisons between two groups were performed using unpaired Student’s *t*-tests, while multiple group comparisons were conducted by one-way analysis of variance (ANOVA) followed by appropriate post hoc tests. Temporal changes in parasitemia rates were evaluated using time-series analysis. Survival times of *Pb*-infected mice were monitored daily using the Kaplan–Meier method and analyzed using the log-rank test. Statistical significance was set at *P* < 0.05.

## Results

### Effects of DSF or TTM treatment on disease progression in *Pb*-infected mice

**Figure 1:**
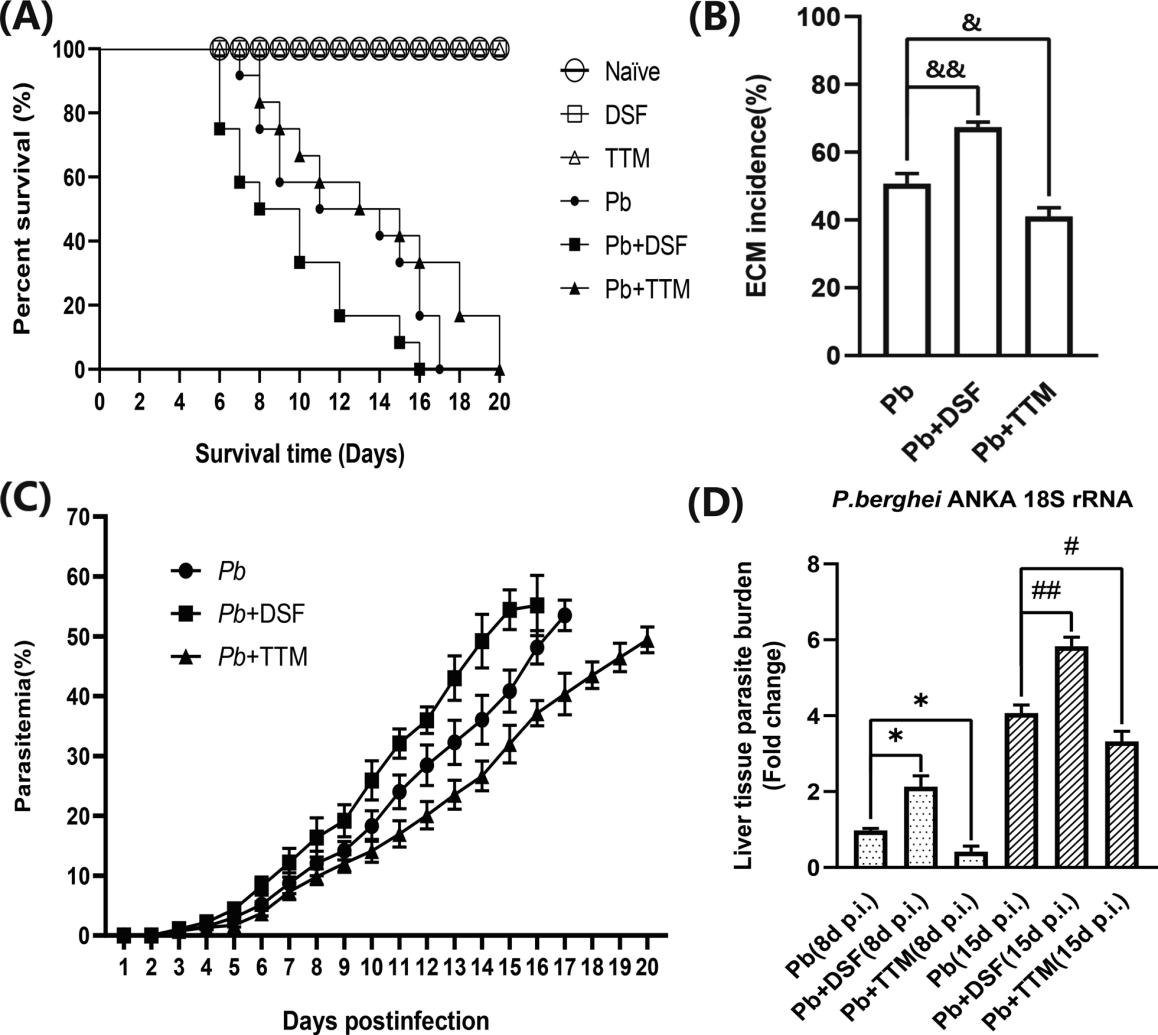
Effects of DSF or TTM treatment on disease progression in *Pb*-infected mice. **A** Survival times of *Pb*-infected mice were monitored daily using the Kaplan–Meier method and analyzed using the log-rank test; **B** the ECM incidence were monitored daily in *Pb*-infected mice from *Pb*-infected, *Pb* + DSF, *Pb* + TTM groups. ^#^*P* < 0.05 and ^##^*P* < 0.01 versus the *Pb*-infected control mice. **C** Peripheral parasitemia was assessed by Giemsa-stained thin blood smears prepared from tail vein blood. Differences among groups were analyzed by time-series analysis; **D** Hepatic parasite burden was evaluated by qPCR detection of *Pb* 18S rRNA in liver tissue at 8 and 15 days post-infection (dpi). *P < 0.05 and **P < 0.01 versus the *Pb*-infected control mice at 8 dpi; ^#^*P* < 0.05 and ^##^*P* < 0.01 versus the *Pb*-infected control mice at 15 dpi. *NS* nonsignificant (*P* > 0.05) relative to naïve mice

As shown in Fig. 1A, B, all uninfected mice from the naïve, DSF-only, and TTM-only groups survived throughout the experiment. *Pb*-infected control mice succumbed between 7 and 17 dpi, with a median survival time of 13 days and an ECM incidence of approximately 50%. The administration of DSF and TTM altered the disease course in *Pb*-infected mice. The overall log-rank test among the three groups (*Pb*, *Pb* + DSF, and *Pb* + TTM) showed a statistically significant difference in survival curve distribution (*P* = 0.0163). However, pairwise post hoc comparisons with Bonferroni correction revealed that the differences between the *Pb* group and each treatment group did not reach statistical significance (*Pb* + DSF versus *Pb*: *P* = 0.0714; *Pb* + TTM versus *Pb*: *P* = 0.1604), whereas a significant difference was observed between the two treatment groups (*Pb* + DSF versus *Pb* + TTM: *P* = 0.0151). Notably, DSF-treated mice tended to succumb earlier (median survival: ~8 days; range: 6–16 dpi; *P* < 0.05) and showed a higher ECM incidence (~67%; *P* < 0.01), whereas TTM-treated mice showed prolonged survival (median: ~15 days; range: 8–20 dpi; *P* < 0.05) and lower ECM incidence (~ 42%; *P* < 0.05). Consistent with these survival outcomes, peripheral parasitemia levels (Fig. 1C) were higher in the *Pb* + DSF group than in the *Pb* group from 5 to 16 dpi, whereas they were lower in the *Pb* + TTM group. Similarly, hepatic parasite burden, assessed by 18S rRNA qPCR (Fig. 1D), was significantly increased in the *Pb* + DSF group at 8 and 15 dpi compared with the *Pb* group (*P* < 0.01), while it was significantly decreased in the *Pb* + TTM group (*P* < 0.01). Together, these data indicated that DSF exacerbated parasitemia and liver parasite burden, whereas TTM reduced both, further supporting the opposing roles of copper modulation in malaria infection.

### Impact of DSF and TTM on liver injury in *Pb*-infected mice

**Figure 2:**
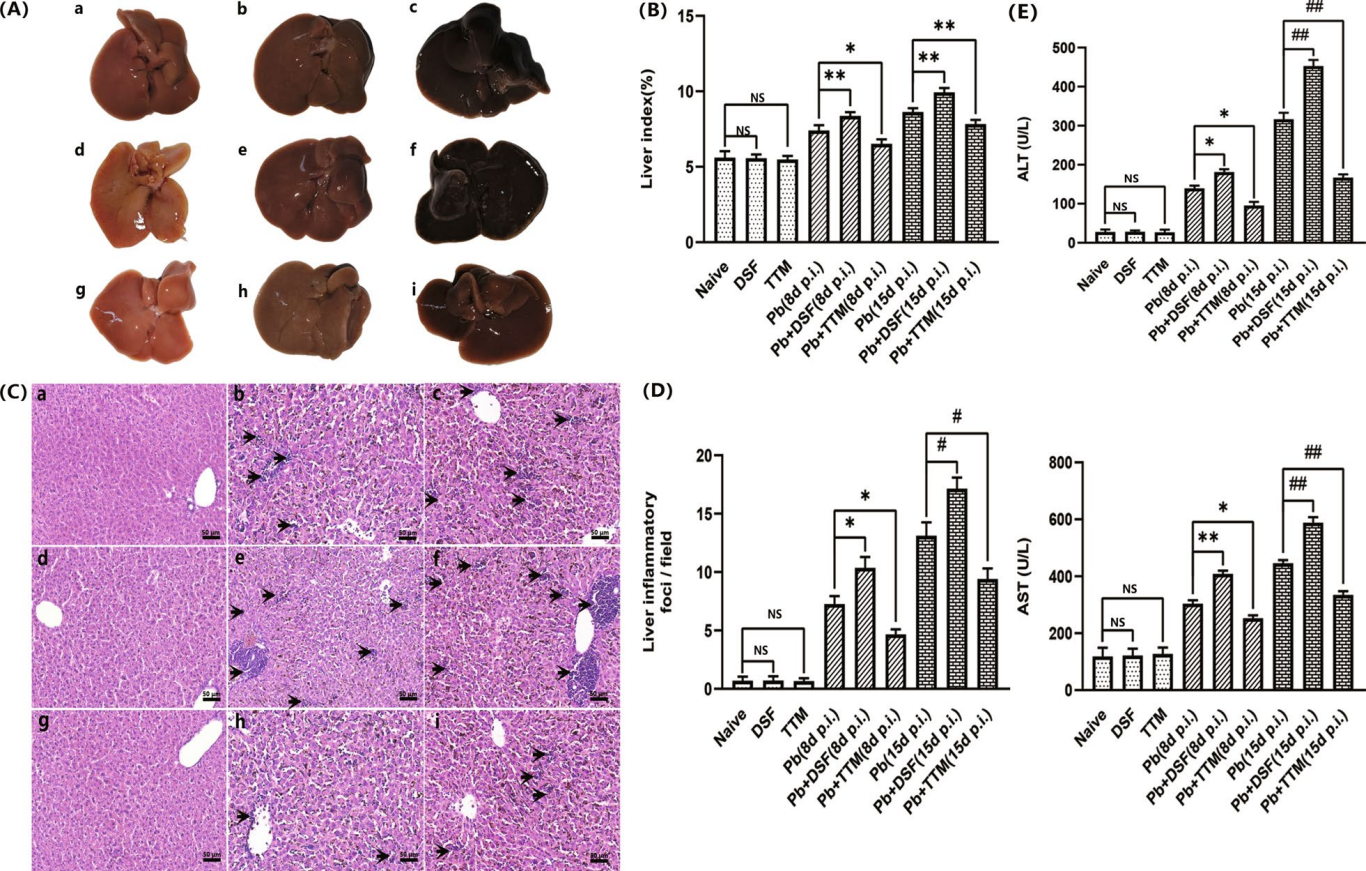
Effects of DSF or TTM treatment on liver morphology, histopathology, and serum liver function biomarkers in *Pb*-infected mice. **A** Representative gross appearance of livers from each experimental group; **B** liver index calculated as liver-to-body weight ratio; **C** histopathological sections of liver tissue stained with hematoxylin and eosin (HE; 200× magnification). **D** Quantitative histopathology scores based on the number of inflammatory foci per visual field. Data are presented as mean ± SD (*n* = 4 ~ 5 mice per group). **E** Serum ALT and AST levels. Group designations: **a** naïve; **b** *Pb*-infected at 8 dpi; **c** *Pb*-infected at 15 dpi; **d** DSF only; **e** *Pb* + DSF at 8 dpi; **f** *Pb* + DSF at 15 dpi; **g** TTM only; **h** *Pb* + TTM at 8 dpi; **i** *Pb* + TTM at 15 dpi. Statistical comparisons were performed using independent-sample *t*-tests (between two groups) or one-way ANOVA (among multiple groups). *P < 0.05, **P < 0.01 versus *Pb*-infected control at 8 dpi; ^#^*P* < 0.05, ^##^*P* < 0.01 versus *Pb*-infected control at 15 dpi; *NS* nonsignificant (*P* > 0.05) versus naïve mice

To assess the role of cuproptosis in hepatic damage during malaria infection, we evaluated liver morphology, liver index, histopathology, and serum markers (ALT and AST) in mice treated with copper ionophore DSF and chelator TTM. Uninfected mice (naïve, DSF-only, and TTM-only groups) exhibited normal liver morphology (pink, smooth, and distinct borders) and minimal histological alterations (Fig. 2). In stark contrast, *Pb* infection alone (*Pb* group) induced profound liver injury by 8 and 15 dpi, characterized by dark red discoloration, significant enlargement (reflected by a marked increase in the liver index,* P* < 0.01 versus naïve), which is a quantitative indicator of hepatomegaly and underlying inflammatory pathology [22], and severe histopathological changes including architectural disarray, dilated sinusoids, extensive inflammatory cell infiltration, and malarial pigment deposition. Critically, modulation of copper death pathways dramatically influenced these outcomes. DSF treatment significantly exacerbated infection-induced liver pathology: livers displayed intensified blackening and further enlargement (< 0.01 versus *Pb* group for liver index), while histology revealed aggravated tissue damage and a marked increase in inflammatory foci at both 8 dpi (*P* < 0.05) and 15 dpi (*P* < 0.05). Conversely, TTM administration substantially mitigated hepatic injury, resulting in visibly lighter liver color, reduced enlargement at 8 dpi (*P* < 0.05) and 15 dpi (*P* < 0.01), attenuated histopathological lesions, and significantly fewer inflammatory spots (*P* < 0.05 versus *Pb* group). Serum biomarker analysis corroborated these findings (Fig. 2E). Infection alone significantly elevated ALT (*P* < 0.01) and AST levels at 8 dpi (*P* < 0.05) and 15 dpi (*P* < 0.01) compared with naïve mice. DSF treatment further amplified these elevations at 8 dpi (*P* < 0.05 for ALT and* P* < 0.01 for AST versus *Pb* group) and 15 dpi (*P* < 0.01 for both ALT and AST versus *Pb* group), indicative of heightened hepatocellular damage. In contrast, TTM treatment significantly reduced serum ALT and AST concentrations compared with the *Pb* group at both 8 dpi (*P* < 0.05) and 15 dpi (*P* < 0.01). Collectively, these data demonstrate that induction of copper death via DSF potently exacerbates, while its inhibition via TTM effectively ameliorates, hepatic injury in *Pb*-infected mice.

### Impact of DSF and TTM on HSC activation and fibrogenesis in *Pb*-infected mice

**Figure 3:**
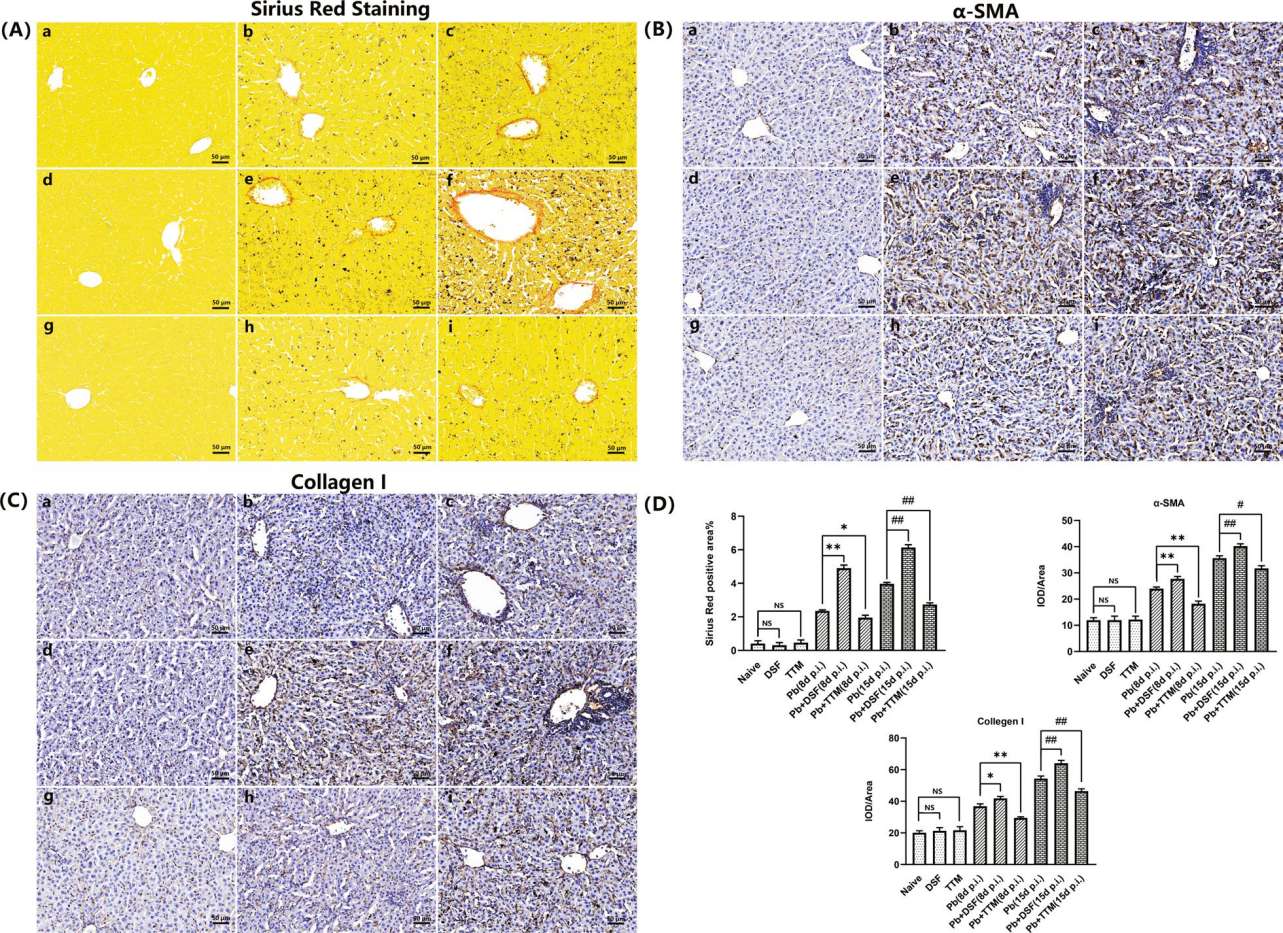
Effects of DSF or TTM treatment on HSC activation and fibrosis in *Pb*-infected mice. **A** Representative Sirius-Red-stained liver sections showing collagen deposition at 8 and 15 days post-infection (200× magnification). **B**, **C** Immunohistochemical staining of α-SMA and Collagen I in liver tissues from each group (200× magnification). **D** Quantitative analysis of Sirius-Red-positive area (%) and integrated optical density (IOD)/area for α-SMA and Collagen I expression, performed using ImageJ FIJI software. All data represent mean ± SD (*n* = 4 ~ 5 mice per group). Group labels: **a** naïve; **b** *Pb*-infected (8 dpi); **c** *Pb*-infected (15 dpi); **d** DSF only; **e** *Pb* + DSF (8 dpi); **f** *Pb* + DSF (15 dpi); **g** TTM only; **h** *Pb* + TTM (8 dpi); **i** *Pb* + TTM (15 dpi). Statistical comparisons were performed using independent sample *t*-tests (two-group) or one-way ANOVA (multiple groups). *P < 0.05, **P < 0.01 versus *Pb*-infected control at 8 dpi; ^#^*P* < 0.05, ^##^*P* < 0.01 versus *Pb*-infected control at 15 dpi; *NS* nonsignificant (*P* > 0.05) versus naïve mice

To investigate the impact of DSF or TTM on HSC activation and consequent fibrogenesis during malaria infection, we assessed collagen deposition and HSC activation markers (α-SMA and Collagen I). Sirius Red staining revealed minimal collagen deposition in livers of uninfected controls (naïve, DSF-only, and TTM-only groups) (Fig. 3). In contrast, *Pb* infection alone (*Pb* group) triggered a significant, time-dependent increase in collagen deposition (*P* < 0.01 versus naïve at 8 and 15 dpi), indicative of progressive fibrogenesis. Crucially, copper death induction via DSF profoundly exacerbated this fibrotic response, significantly elevating collagen accumulation at both 8 and 15 dpi compared with *Pb* group (*P* < 0.01). Conversely, TTM-mediated copper chelation markedly suppressed collagen deposition in infected mice (*P* < 0.05 at 8 dpi, *P* < 0.01 at 15 dpi versus *Pb* group). Immunohistochemical analysis further demonstrated that infection significantly upregulated hepatic expression of the HSC activation marker α-SMA and the key fibrillar collagen Collagen I (*P* < 0.01 versus naïve). DSF treatment amplified this response, inducing significantly higher α-SMA (*P* < 0.01) and Collagen I (*P* < 0.05 at 8 dpi, *P* < 0.01 at 15 dpi) levels than the *Pb* group at both timepoints. In stark opposition, TTM administration substantially reduced the expression of α-SMA (*P* < 0.01 at 8 dpi, *P* < 0.05 at 15 dpi) and Collagen I (*P* < 0.01). Collectively, these data demonstrate that induction of copper death by DSF promotes HSC activation and drives collagen deposition, whereas TTM-mediated copper chelation effectively suppresses HSC-driven fibrogenesis in the malaria-infected liver.

### Impact of DSF and TTM on hepatic copper overload and key cuproptosis-related protein expression in Pb-infected mice

**Figure 4:**
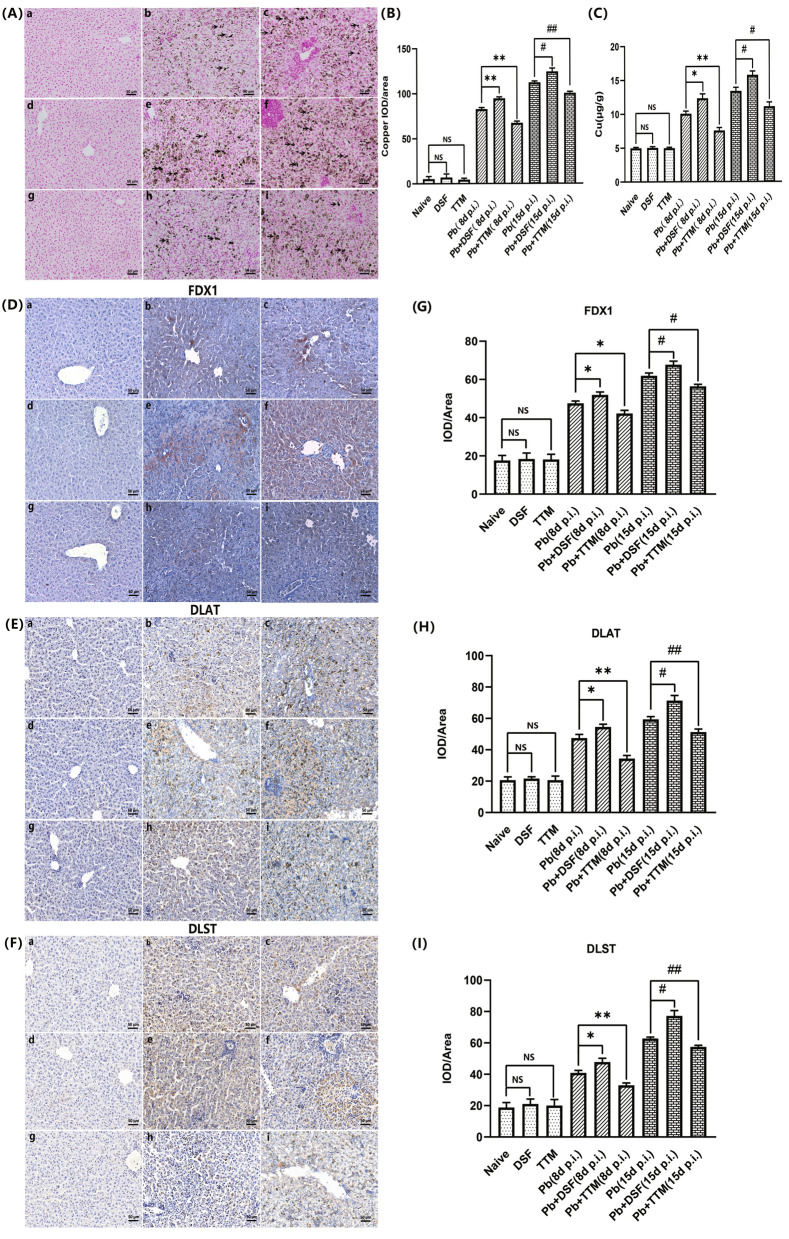
Hepatic copper accumulation and cuproptosis-related proteins expression in *Pb*-infected mice following DSF or TTM treatment. **A** Representative photomicrographs of liver sections stained with rubeanic acid to detect copper deposits (dark brown, indicated by arrows; 200× magnification). Group labels: **a** naïve; **b** *Pb*-infected (8 dpi); **c** *Pb*-infected (15 dpi); **d** DSF only; **e** *Pb* + DSF (8 dpi); **f** *Pb* + DSF (15 dpi); **g** TTM only; **h** *Pb* + TTM (8 dpi); **i** *Pb* + TTM (15 dpi). **B** Quantification of copper deposits expressed as integrated optical density (IOD) per area, analyzed using ImageJ FIJI software; **C** Hepatic copper concentration measured by inductively coupled plasma mass spectrometry (ICP-MS). **D**–**F** Representative immunohistochemical staining images showing expression of cuproptosis-related proteins (FDX1, DLAT, and DLST) in liver tissues from different groups (200× magnification); group labels: **a** naïve; **b** *Pb*-infected (8 dpi); **c** *Pb*-infected (15 dpi); **d** DSF only; **e** *Pb* + DSF (8 dpi); **f** *Pb* + DSF (15 dpi); **g** TTM only; **h** *Pb* + TTM (8 dpi); **i** *Pb* + TTM (15 dpi). **G**–**I** Quantitative analysis of protein expression presented as integrated optical density (IOD) per area, determined using ImageJ FIJI software. Data are presented as mean ± SD (*n* = 4 ~ 5 mice per group). Statistical comparisons were performed using independent sample *t*-tests (two groups) or one-way ANOVA (multiple groups). *P < 0.05, **P < 0.01 versus *Pb*-infected control at 8 dpi; ^#^*P* < 0.05, ^##^*P* < 0.01 versus *Pb*-infected control at 15 dpi; *NS* nonsignificant (*P* > 0.05) versus naïve mice

To define the role of copper dysregulation and cuproptosis in malaria-induced liver injury, hepatic copper accumulation and key cuproptosis-related protein expression were analyzed. Rubeanic acid (RAC) staining revealed minimal copper salt deposition in livers of uninfected controls (naïve, DSF, TTM) (Fig. 4A, B). In contrast, *Pb* infection (*Pb* group) provoked significant, time-dependent hepatic copper accumulation at 8 and 15 dpi (*P* < 0.01 versus naïve). Crucially, DSF treatment dramatically exacerbated copper overload in infected mice, elevating deposition further at both timepoints (*P* < 0.01 at 8 dpi, *P* < 0.05 at 15 dpi). Conversely, TTM administration effectively suppressed copper accumulation (*P* < 0.01 versus *Pb* group). Inductively coupled plasma mass spectrometry (ICP-MS) quantification of total hepatic copper confirmed these RAC staining patterns (Fig. 4C). Immunohistochemical analysis demonstrated that *Pb* infection significantly upregulated expression of cuproptosis effector proteins FDX1, DLAT, and DLST in hepatocytes at 8 and 15 dpi (*P* < 0.01 versus naïve) (Fig. 4D–I). DSF co-treatment amplified this response, inducing substantially higher FDX1 (*P* < 0.05), DLAT (*P* < 0.05), and DLST (*P* < 0.05) expression versus the *Pb* group. Notably, TTM intervention markedly attenuated the infection-driven upregulation of FDX1 (*P* < 0.05), DLAT (*P* < 0.01), and DLST (*P* < 0.01). Collectively, these findings demonstrate that *Pb* infection induces hepatic copper overload and activates the cuproptosis execution pathway (FDX1/DLAT/DLST), which is potentiated by DSF-mediated copper accumulation and mitigated by TTM’s copper-chelating action, directly linking copper-driven cell death to malaria-associated liver injury.

### Impact of DSF and TTM on cuproptosis-related protein expression in HSCs of *Pb*-infected mice

**Figure 5:**
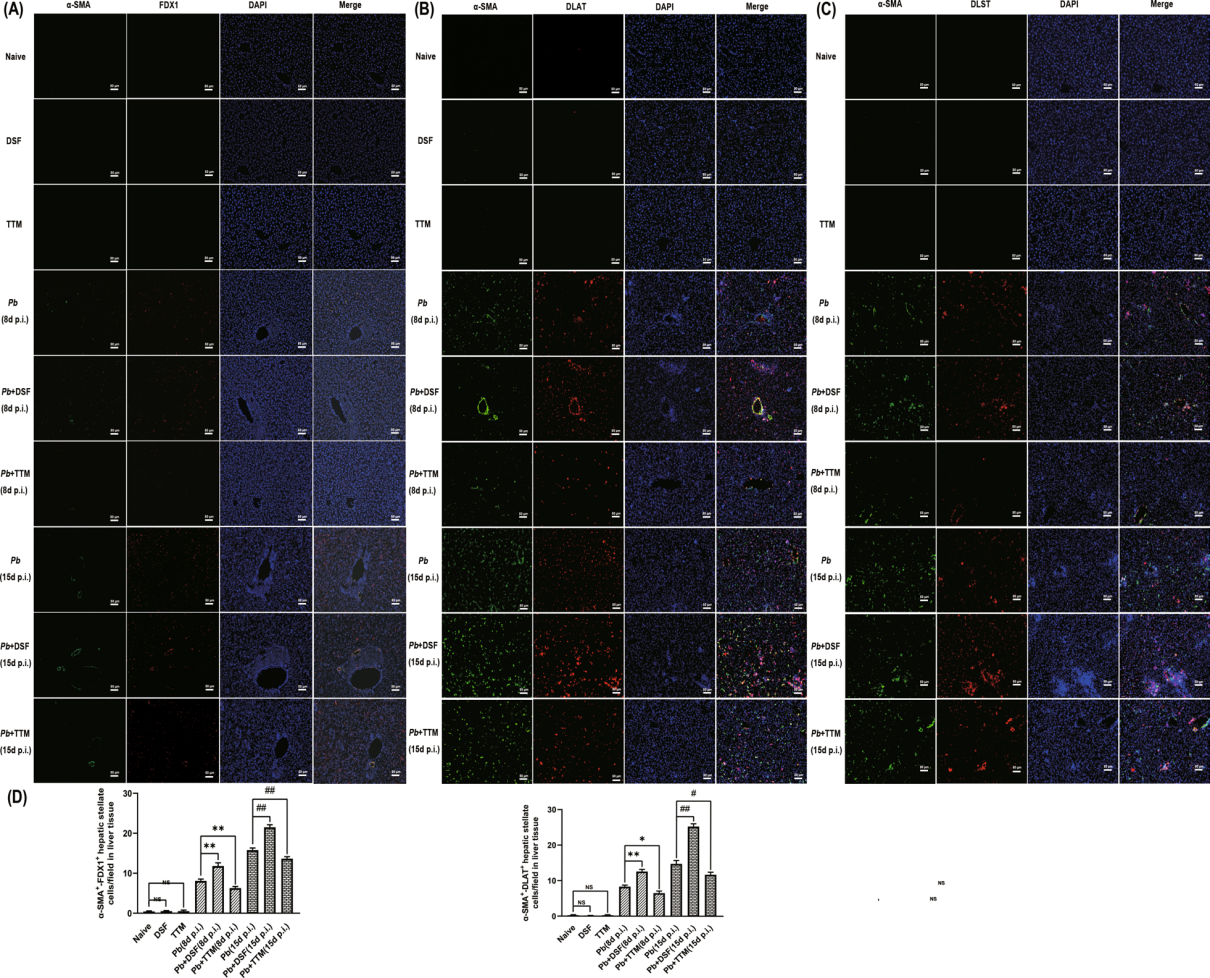
Effects of DSF or TTM on the expression of the cuproptosis-associated marker (FDX1, DLAT, and DLST) in activated HSCs in response to *Pb*-induced acute liver injury. **A**–**C** Representative double immunofluorescence staining of liver sections (200×) showing α-SMA⁺ HSCs (green); cuproptosis-related proteins FDX1, DLAT, and DLST (red); and their co-localization (yellow) indicating activated HSCs undergoing cuproptosis. Group labels: **a** naïve; **b** *Pb*-infected (8 dpi); **c** *Pb*-infected (15 dpi); **d** DSF only; **e** *Pb* + DSF (8 dpi); **f** *Pb* + DSF (15 dpi); **g** TTM only; **h** *Pb* + TTM (8 dpi); **i** *Pb* + TTM (15 dpi). **D** Quantification of FDX1⁺-α-SMA⁺, DLAT⁺-α-SMA⁺, and DLST⁺-α-SMA⁺ double-positive HSCs from 20 randomly selected fields per mouse. Data are presented as mean ± SD (*n* = 4 ~ 5 mice per group). Statistical comparisons were performed using independent sample *t*-tests (between two groups) or one-way ANOVA (among multiple groups). *P < 0.05, **P < 0.01 versus *Pb*-infected control at 8 dpi; ^#^*P* < 0.05, ^##^*P* < 0.01 versus *Pb*-infected control at 15 dpi; *NS* nonsignificant (*P* > 0.05) versus naïve mice

Double immunofluorescence analysis targeting α-SMA (marking activated HSCs) and key cuproptosis effectors (FDX1, DLAT, and DLST) revealed minimal co-localization in the livers of uninfected control mice (naïve, DSF, and TTM groups) (Fig. 5). In stark contrast, *Pb* infection alone (*Pb* group) induced a significant upregulation of FDX1⁺/α-SMA⁺, DLAT⁺/α-SMA⁺, and DLST⁺/α-SMA⁺ co-labeled HSC at both 8 and 15 dpi compared with naïve controls (*P* < 0.01 for all comparisons). Pharmacological modulation of copper availability profoundly altered this response. Treatment with the copper ionophore DSF (*Pb* + DSF group) significantly exacerbated the infection-induced effect, further increasing the numbers of FDX1⁺/α-SMA⁺ (*P* < 0.01), DLAT⁺/α-SMA⁺ (*P* < 0.01) and DLST⁺/α-SMA⁺ (*P* < 0.01) HSCs relative to the *Pb* group. Conversely, copper chelation with TTM (*Pb* + TTM group) markedly suppressed the expression of these cuproptosis markers in activated HSCs, resulting in significantly reduced numbers of FDX1⁺/α-SMA⁺ (*P* < 0.01), DLAT⁺/α-SMA⁺ (*P* < 0.05), and DLST⁺/α-SMA⁺ (*P* < 0.01) cells compared with the *Pb* group. Collectively, these data demonstrate that *P. berghei* infection triggers the upregulation of mitochondrial cuproptosis effectors within activated hepatic stellate cells, a pathological process amplified by copper elevation via DSF and attenuated by copper depletion via TTM during malarial liver injury.

### Impact of apoptosis in liver tissue of mice with malaria-associated acute liver injury

**Figure 6:**
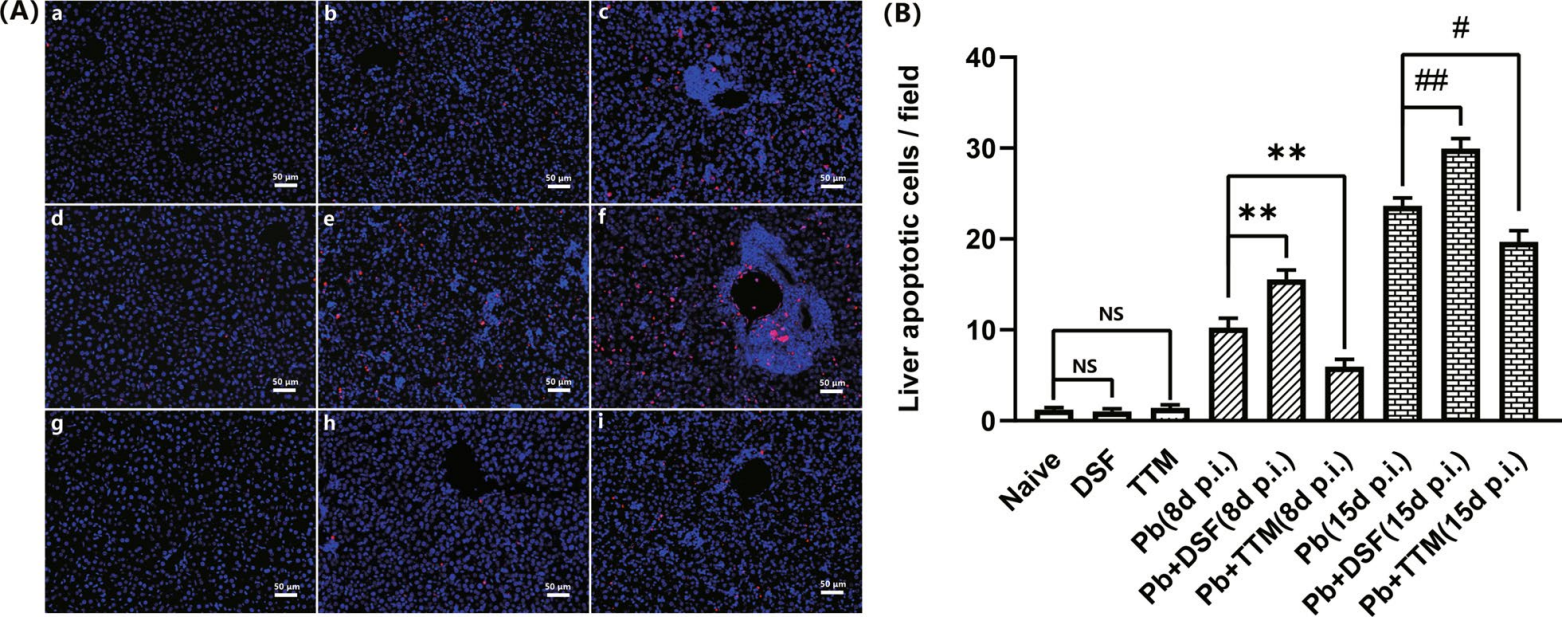
Hepatocyte apoptosis in malaria-associated acute liver injury following DSF or TTM treatment. **A** Representative TUNEL-stained liver sections (200×) showing apoptotic cells. Group labels: **a** naïve; **b** *Pb*-infected (8 dpi); **c** *Pb*-infected (15 dpi); **d** DSF only; **e** *Pb* + DSF (8 dpi); **f** *Pb* + DSF (15 dpi); **g** TTM only; **h** *Pb* + TTM (8 dpi); **i** *Pb* + TTM (15 dpi). **B** Apoptotic index quantified as the number of TUNEL-positive cells per field across 20 random visual fields per mouse. Data are presented as mean ± SD (*n* = 4 ~ 5 mice per group). Statistical comparisons were performed using independent sample *t*-tests (between two groups) or one-way ANOVA (among multiple groups). *P < 0.05, **P < 0.01 versus *Pb*-infected control at 8 dpi; ^#^*P* < 0.05, ^##^*P* < 0.01 versus *Pb*-infected control at 15 dpi; *NS* nonsignificant (*P* > 0.05) versus naïve mice

We investigated the effects of DSF and TTM treatment on hepatocyte apoptosis in malaria-associated acute liver injury. As shown in Fig. 6, few or no TUNEL-positive staining cells were observed in liver tissue of naïve, DSF-only and TTM-only uninfected mice. Compared with naïve group, the number of apoptotic cells in liver tissue of *Pb* group increased significantly at 8 and 15 days after infection with *Pb* (*P* < 0.01). Compared with *Pb* group, apoptotic cells in liver tissue of infected mice treated with DSF increased significantly (*P* < 0.01), while apoptotic cells in liver tissue of infected mice treated with TTM decreased significantly (*P* < 0.01 at 8 dpi, *P* < 0.05 at 15 dpi). These data suggest that DSF or TTM treatment increases or decreases the number of apoptotic cells in liver tissue, respectively, during liver injury in malaria-infected mice.

### Impact of DSF and TTM on profibrotic and antifibrotic cytokines in liver tissues of *Pb*-infected mice

**Figure 7:**
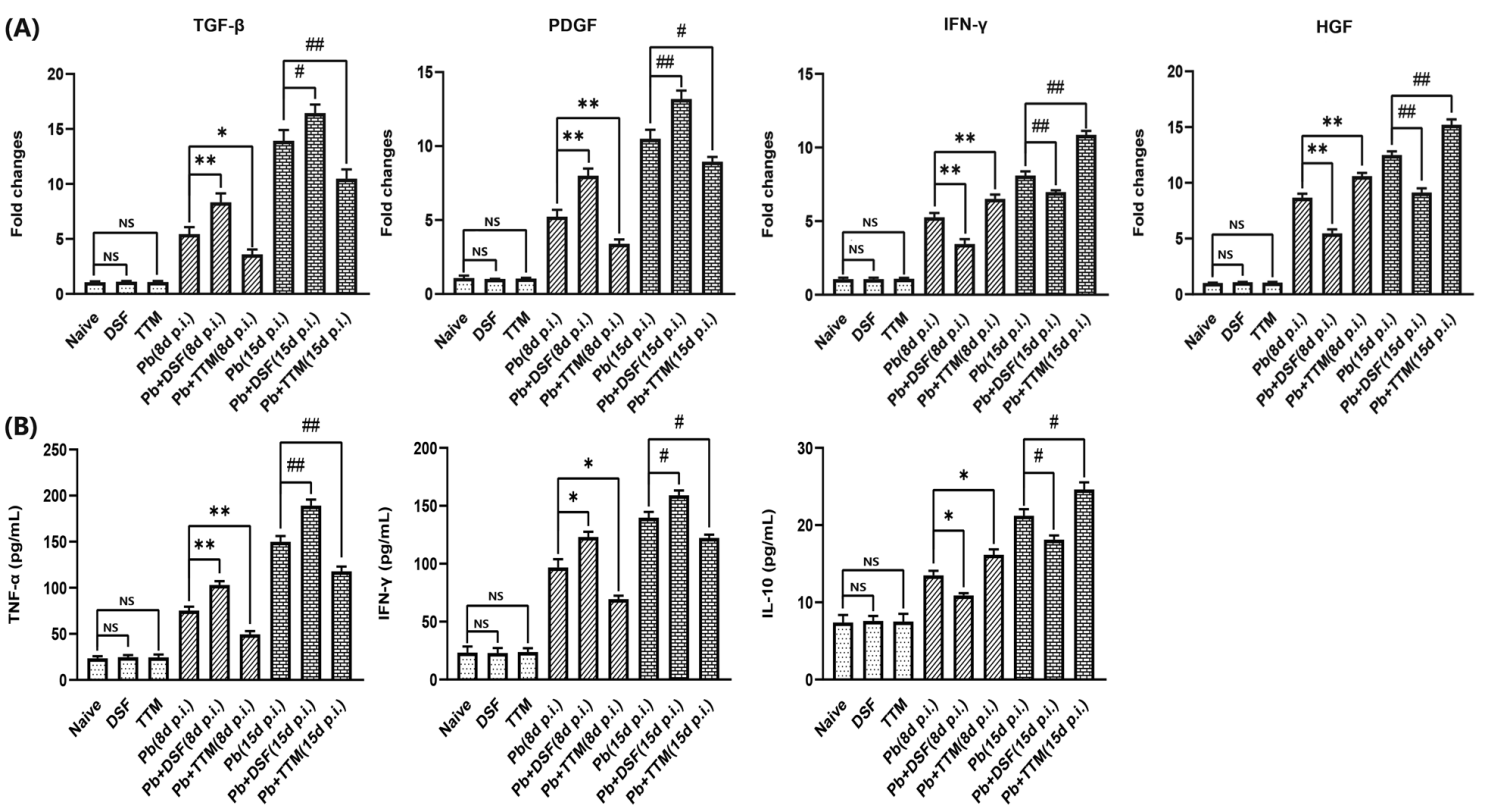
Effects of DSF and TTM on mRNA expression of HSC activation and liver fibrosis markers, as well as serum inflammatory cytokines, in malaria-associated acute liver injury. **A** Total RNA was isolated from liver tissues of experimental mice, and mRNA expression levels of TGF-β, PDGF, IFN-γ, and HGF were determined by quantitative PCR using the 2^−ΔΔCT^ method. **B** Serum levels of TNF-α, IFN-γ, and IL-10 were analyzed by ELISA kit. Data are shown as mean ± SD (*n* = 4 mice per group). Statistical comparisons were performed using an independent sample *t*-test (for two-group comparisons) or one-way ANOVA (for multigroup comparisons). *P < 0.05, **P < 0.01 versus *Pb*-infected control at 8 dpi; ^#^*P* < 0.05, ^##^*P* < 0.01 versus *Pb*-infected control at 15 dpi; *NS* nonsignificant (*P* > 0.05) versus naïve mice

qPCR analysis revealed that *Pb* infection alone triggered a significant induction of key cytokines within the liver microenvironment compared with uninfected controls (*Pb* group versus naïve: TGF-β, PDGF, IFN-γ, and HGF all *P* < 0.01; Fig. 7A). While DSF or TTM administration alone to uninfected mice exerted no significant effect on baseline cytokine mRNA levels (*P* > 0.05), their impact during infection was profound and divergent. Treatment with the copper ionophore DSF (*Pb* + DSF group) significantly potentiated the expression of profibrotic mediators TGF-β (*P* < 0.01 at 8 dpi, * P* < 0.05 at 15 dpi) and PDGF (*P* < 0.01 at both timepoints) relative to infected, untreated controls (*Pb* group). Concurrently, DSF suppressed mRNA levels of antifibrotic cytokines IFN-γ (*P* < 0.01) and HGF (*P* < 0.01 at both timepoints). Conversely, copper chelation via TTM (*Pb* + TTM group) significantly attenuated the infection-driven upregulation of TGF-β (*P* < 0.05 at 8 dpi,* P* < 0.01 at 15 dpi) and PDGF (*P* < 0.01 at 8 dpi,* P* < 0.05 at 15 dpi), while enhancing the expression of IFN-γ (*P* < 0.01) and HGF (*P* < 0.01) compared with the *Pb* group at both 8 and 15 dpi. These data demonstrate that copper availability critically shapes the hepatic cytokine profile during malarial infection, with DSF promoting a profibrotic imbalance favoring HSC activation and TTM fostering an antifibrotic environment that constrains HSC activation.

### Impact of DSF and TTM on systemic inflammatory responses in *Pb*-infected mice

To assess the systemic inflammatory response, serum levels of TNF-α, IFN-γ, and IL-10 were measured by ELISA (Fig. 7B). Compared with naïve mice, *Pb* infection alone significantly elevated the levels of TNF-α, IFN-γ, and IL-10 at both 8 and 15 dpi (*P* < 0.01 versus naïve). Compared with the *Pb* group, DSF-treated mice exhibited significantly higher levels of TNF-α at both 8 and 15 dpi (*P* < 0.01), as well as significantly increased IFN-γ levels at both timepoints (*P* < 0.05). Conversely, DSF treatment significantly reduced IL-10 levels at 8 and 15 dpi (*P* < 0.05 versus *Pb* group). In contrast, compared with the *Pb* group, TTM-treated mice showed significantly lower TNF-α levels at both 8 and 15 dpi (*P* < 0.01), along with significantly reduced IFN-γ levels at both timepoints (*P* < 0.05), while IL-10 levels were significantly elevated at both timepoints (*P* < 0.05 versus *Pb* group).

### Impact of DSF-CuCl_2_ and TTM-CuCl_2_ on cuproptosis and HSC activation in iRBC-stimulated HSCT6 cells

**Figure 8:**
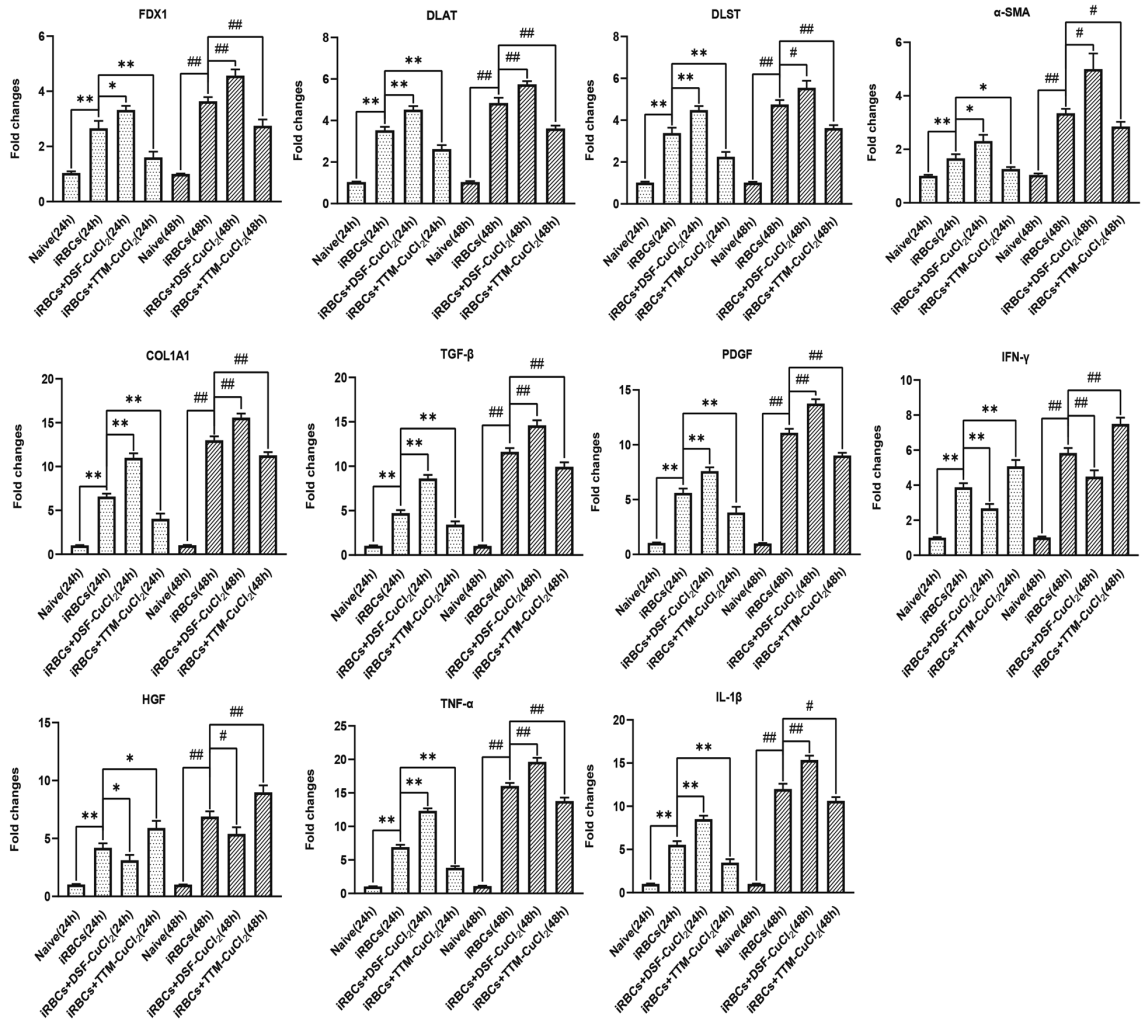
Effects of DSF-CuCl_2_ and TTM-CuCl_2_ on mRNA expression of cuproptosis and fibrotic markers in iRBC-stimulated HSCT6 cells. HSCT6 cells were pretreated with 1.0 × 10^5^ iRBCs for 6 h, followed by treatment with PBS, DSF-CuCl_2_, or TTM-CuCl_2_ for 24 or 48 h. The mRNA expression levels of FDX1, DLAT, DLST, α-SMA, COL1A1, TGF-β, PDGF, IFN-γ, HGF, TNF-α, and IL-1β were quantified by qPCR method and normalized to GAPDH. Data are presented as mean ± SD from four independent experiments showing consistent results. *P < 0.05, **P < 0.01 versus iRBC-stimulated HSCT6 cells at 24 h; ^#^*P* < 0.05, ^##^*P* < 0.01 versus iRBC-stimulated HSCT6 cells at 48 h

To investigate the role of copper homeostasis in hepatic stellate cell responses to *P. berghei* infection in vitro, HSCT6 cells were stimulated with *Pb*-iRBCs and concurrently treated with DSF-CuCl_2_ or TTM-CuCl_2_ for 24 or 48 h (Fig. 8). qPCR analysis revealed that stimulation with non-parasitized RBCs did not significantly alter cytokine expression compared with unstimulated controls. However, *Pb*-iRBCs stimulation significantly upregulated mRNA levels of key cuproptosis regulators (FDX1, DLAT, and DLST) and markers of stellate cell activation (α-SMA and COL1A1), fibrosis (TGF-β and PDGF), and proinflammatory cytokines (TNF-α and IL-1β), while concurrently downregulating antifibrotic cytokines (IFN-γ and HGF) at both timepoints compared with the blank group (*P* < 0.01). DSF-CuCl_2_ treatment markedly potentiated these iRBC-induced effects. It significantly enhanced the expression of FDX1 (*P* < 0.05 and *P* < 0.01), DLAT (*P* < 0.01), DLST (*P* < 0.01 and *P* < 0.05), α-SMA (*P* < 0.05), COL1A1 (*P* < 0.01), TGF-β (*P* < 0.01), PDGF (*P* < 0.01), TNF-α (*P* < 0.01), and IL-1β (*P* < 0.01), while further suppressing IFN-γ (*P* < 0.01) and HGF (*P* < 0.05) mRNA levels. Conversely, TTM-CuCl_2_ treatment effectively attenuated the iRBC-driven changes. It significantly reduced the elevated mRNA levels of FDX1 (*P* < 0.01), DLAT (*P* < 0.01), DLST (*P* < 0.01), α-SMA (*P* < 0.05), COL1A1 (*P* < 0.01), TGF-β (*P* < 0.01), PDGF (*P* < 0.01), TNF-α (*P* < 0.01), and IL-1β (*P* < 0.01 and *P* < 0.05), while restoring the expression of the antifibrotic mediators IFN-γ (*P* < 0.01) and HGF (*P* < 0.05 and *P* < 0.01). Collectively, these findings demonstrate that DSF-CuCl_2_ exacerbates iRBC-induced cuproptosis and promotes a profibrogenic phenotype in hepatic stellate cells in vitro, whereas TTM-CuCl_2_ mitigates these effects by reducing cuproptosis and suppressing stellate cell activation.

## Discussion

Acute liver injury represents a commonly observed pathological feature in severe malaria, characterized histopathologically by hepatocyte swelling and necrosis, HSC activation, deposition of malaria pigment, Kupffer cell hyperplasia, and inflammatory cell infiltration [3]. HSCs, which account for ~15% of all liver cells, are well-established drivers of fibrosis in chronic liver diseases such as viral hepatitis [25]. However, growing evidence indicated HSC activation and fibrogenic responses also occurred in the context of acute hepatic damage, including severe malaria infection and acute liver failure [26–28]. Experimental malaria models had successfully recapitulated these pathological changes, demonstrating that HSC activation exacerbated liver fibrosis through signaling pathways such as Hedgehog and Yes-associated protein [28, 29]. Copper, an essential trace element absorbed in the intestine and predominantly metabolized and stored in the liver—the organ with the highest copper content in the body [10]—was known to accumulate under pathological conditions such as Wilson’s disease, where it promoted hepatocyte injury, HSC activation, and fibrosis [15]. Notably, our recent studies revealed marked hepatic copper accumulation in mice suffering from malaria-associated acute liver injury. This observation was consistent with prior reports of dysregulated cuproptosis-related gene expression in other models of acute liver injury [30]. We therefore postulated that copper overload and the subsequent induction of cuproptosis contributed to the pathogenesis of malaria-induced acute liver injury, specifically through the activation of HSCs. In support of this hypothesis, our experimental data confirmed the coexistence of hepatic copper accumulation and cuproptosis in mice with malaria-induced liver injury. More importantly, pharmacological modulation established a causal relationship: DSF, a known inducer of cuproptosis, aggravated HSC activation, liver injury, and fibrotic responses. In contrast, treatment with the copper chelator TTM attenuated cuproptosis, suppressed HSC activation, and thereby ameliorated both liver injury and fibrosis. Taken together, these results identified copper accumulation-induced cuproptosis as a novel pathogenic mechanism that promotes HSC activation and exacerbated liver damage in murine malaria. Our findings may underscore the potential therapeutic value of targeting cuproptosis or modulating copper homeostasis to alleviate acute liver injury in severe malaria.

Disruption of copper homeostasis and subsequent copper accumulation-induced cuproptosis have been increasingly implicated in a range of hepatic pathologies, including hepatocellular carcinoma, Wilson’s disease, and metabolic dysfunction-associated fatty liver disease [31–34]. In the context of malaria, copper bioavailability was known to critically influence *P. falciparum* parasite development within erythrocytes, as demonstrated by the ability of copper chelators such as neocuprine to inhibit the parasite’s transition from ring to trophozoite stages [35, 36]. Although clinical studies presented conflicting data regarding serum copper levels in malaria patients—with some cohorts showing a decrease correlated with disease severity [37] and others reporting elevated levels [38]—the role of intrahepatic copper accumulation in malaria-associated acute liver injury had remained largely unexplored. In this study, we identified significant hepatic copper accumulation and upregulation of key cuproptosis-related mediators (FDX1, DLAT, and DLST) in a murine model of malaria-induced acute liver injury, reflecting patterns of cuproptosis-gene dysregulation previously observed in metabolic fatty liver disease [33, 39]. Importantly, pharmacological modulation of copper homeostasis established a direct causal relationship: DSF, which functions as a copper ionophore to promote intracellular copper delivery and cuproptosis [40], aggravated hepatic copper overload, elevated FDX1/DLAT/DLST expression, increased parasite burden, and exacerbated liver histopathology. In contrast, treatment with the copper chelator TTM attenuated these effects [41], consistent with reports that FDX1 was elevated in copper-dependent liver injury and that TTM suppressed cuproptosis-related gene expression [42, 43]. These findings were further validated in vitro using iRBC-stimulated HSCT6 cells, which confirmed that DSF upregulated, whereas TTM downregulated, FDX1/DLAT/DLST mRNA levels. Together, our results established copper overload-driven cuproptosis as a novel pathological mechanism that exacerbated acute liver injury in murine malaria, potentially offering a therapeutic target distinct from copper’s recognized role in parasite development.

Having established that copper overload drove cuproptosis and HSC activation, we next considered whether iron dysregulation, which was another metal-related pathway implicated in malaria pathology, might intersect with our findings. Beyond copper dysregulation, perturbations in iron homeostasis represented a parallel and potentially synergistic mechanism in malaria-associated liver injury. Hemozoin, the crystalline byproduct of hemoglobin digestion by *Plasmodium*, accumulated abundantly in the liver during infection and acted as a damage-associated molecular pattern that triggered inflammatory responses and compromised cellular viability [44, 45]. Recent evidence demonstrated that hemozoin uptake by Kupffer cells drove mitochondrial superoxide production, lipid peroxidation, and intracellular free iron accumulation, which ware characteristic features of ferroptosis, leading to macrophage depletion and exacerbation of liver injury [45, 46]. A particularly intriguing link between iron and copper metabolism in malaria had recently been elucidated through high-precision isotopic analysis. Costas-Rodríguez and colleagues demonstrated that *Plasmodium* infection induced marked hepatic iron overload in mice, accompanied by distinct isotopic shifts in copper consistent with ceruloplasmin-mediated copper mobilization into the bloodstream to support iron trafficking [47]. This finding raised the possibility that the hepatic copper accumulation we observed, as the central trigger of cuproptosis, may be mechanistically coupled to systemic iron redistribution during malaria. The convergence of these two metal-driven pathways at the level of mitochondrial homeostasis was especially noteworthy: mitochondria served both as primary targets of iron-overload-induced oxidative injury and as the central execution site of cuproptosis [48]. On this basis, we hypothesize that hemozoin deposition and subsequent iron overload represent an initiating insult in malaria-associated liver injury, whereas copper accumulation and cuproptosis amplify and perpetuate this damage. Together, these interconnected metal dysregulation pathways may cooperatively drive HSC activation and fibrogenesis through overlapping mitochondrial stress signals. Future studies exploring the combined therapeutic targeting of iron and copper homeostasis, particularly through dual chelation strategies, may offer enhanced hepatoprotection in severe malaria.

HSCs, which are resident perisinusoidal cells responsible for vitamin A storage in the healthy liver, undergo activation into myofibroblast-like phenotypes in response to inflammatory signals derived from injured hepatocytes and immune cells during liver pathology [26, 49]. Once activated, HSCs drove fibrogenesis through excessive deposition of extracellular matrix (ECM) [50]. Copper dysregulation had previously been implicated in promoting hepatocyte injury, HSC activation, and ECM accumulation in chronic liver settings [51, 52]. In an experimental model of malaria-associated acute liver injury, we now demonstrated that pharmacological modulation of copper homeostasis directly influences HSC-mediated fibrosis. Specifically, DSF, a copper ionophore known to induce cuproptosis, significantly increased the number of activated HSCs (α-SMA⁺/FDX1⁺ cells) and enhanced collagen deposition. In contrast, the copper chelator TTM attenuated both HSC activation and fibrotic accumulation. Correspondingly, in iRBC-stimulated HSCT6 cells, treatment with DSF + CuCl_2_ led to upregulation of profibrogenic mediators (α-SMA, TGF-β, and PDGF mRNA) and downregulation of antifibrotic cytokines (IFN-γ and HGF mRNA), whereas TTM reversed these expression alterations. Importantly, the correlation observed in vivo between upregulation of the cuproptosis markers FDX1 and DLST and expansion of α-SMA⁺ HSCs established cuproptosis as a key mechanism driving HSC activation in this setting. Therefore, DSF exacerbated and TTM alleviated malaria-associated liver fibrosis, respectively, by promoting or inhibiting cuproptosis-induced HSC activation—revealing a druggable pathway for mitigating parasite-induced fibrotic pathology. To distinguish the effects of pharmacological copper modulation from infection-induced pathology, we included uninfected control groups receiving DSF or TTM alone. Throughout all experiments, these groups showed no significant differences from naïve controls, confirming that DSF or TTM administration alone does not induce hepatic pathology in the absence of *Pb* infection.

An important observation in our study was that despite continuous TTM treatment, hepatic inflammation and collagen deposition still progressed from 8 to 15 dpi, albeit to a significantly lesser extent than in untreated infected controls. Although this may appear inconsistent with a copper-dependent mechanism, it aligned with the well-established concept of a “point of no return” in HSC activation. HSC activation was a multistage process comprising an “initiation phase” and a subsequent “perpetuation phase” [53, 54]. During initiation, paracrine signals from injured hepatocytes and immune cells—including copper-induced cuproptosis signals—triggered the initial transdifferentiation of quiescent HSCs [53]. TTM targeted this upstream copper overload, effectively reducing de novo HSC activation, which explained the marked improvement observed in TTM-treated mice relative to untreated infected controls. However, once HSCs had transitioned to fully activated myofibroblasts, they entered the perpetuation phase, characterized by autocrine production of profibrogenic cytokines such as TGF-β1 and PDGF, which sustained fibrogenic activity even after the original trigger is removed [55–57]. This was supported by our qPCR data, showing that TTM significantly but incompletely reduced TGF-β and PDGF expression. Moreover, accumulating evidence indicated that HSC activation involved profound epigenetic remodeling, including DNA methylation and histone modifications, which conferred a stable “activation memory” that locked cells into a sustained fibrogenic state [57, 58]. These epigenetic changes activated HSCs to maintain their phenotype independently of the initial stimulus [58, 59]. This concept of a self-perpetuating, epigenetically stabilized activated state directly explains why copper chelation alone, while effectively limiting the amplitude of the fibrotic response, cannot completely halt its temporal progression once a substantial pool of activated HSCs had been established. Importantly, this pattern of partial but significant efficacy was consistent with the known limitations of upstream-targeted antifibrotic therapies. As noted in a recent perspective on antifibrotic drug development, even promising agents often showed incomplete reversal of established fibrosis, prompting debate over whether “fibrosis reversal should be the only goal” [60]. Studies on TTM in other fibrosis models, such as bile duct ligation-induced liver fibrosis, had similarly reported that TTM significantly attenuates injury and collagen deposition but did not completely prevent disease progression [61]. Parallel observations had also been made in other models of infection-induced pathology, in which therapeutic intervention significantly improves outcomes at all timepoints yet disease continues to progressively worsen over time [62]. Thus, our findings with TTM were consistent with both the fundamental biology of HSC activation and the broader experience in antifibrotic therapy development.

Apoptosis, a programmed cell death mechanism essential for tissue homeostasis, played a pivotal role in the pathogenesis of liver diseases by amplifying inflammatory responses and promoting fibrosis [63, 64]. In liver injury, activation of the NF-κB pathway recruited inflammatory cells and stimulated the release of cytokines such as IL-6, TNF-α, and TGF-β, which in turn exacerbated inflammation and fibrogenesis. Notably, the level of hepatocyte apoptosis was correlated with disease severity and fibrosis progression in conditions such as alcoholic liver disease [64]. Importantly, hepatocyte apoptosis not only directly activated HSCs, but was also intensified through a pathologic feedback loop involving inflammation and fibrosis [65]. Inhibition of apoptosis—for instance, via blockade of the Fas system—had been shown to attenuate fibrosis in models such as pulmonary fibrosis [66]. Activated HSCs further drove hepatocyte apoptosis through multiple mechanisms, including reactive oxygen species (ROS) production mediated by NOX1/NOX4 (whose inhibition reduces both apoptosis and fibrosis [67]) and the secretion of proinflammatory cytokines such as TNF-α and IL-1β. In the context of liver injury, these cytokines can bind to death receptors such as TNFR1 on hepatocytes and potentially activate the caspase pathway, thereby promoting apoptosis [68]. In our experimental model of malaria-associated acute liver injury, extensive hepatocyte apoptosis was observed. This apoptotic response was significantly aggravated by DSF, which promoted cuproptosis and HSC activation, but was alleviated by the copper chelator TTM. In vitro studies using HSCT6 cells demonstrated that DSF promoted HSC activation by inducing cuproptosis, which was accompanied by upregulation of TNF-α and IL-1β mRNA levels. This suggested that activated HSCs represent a potential source of these proinflammatory mediators. The progression of malaria was closely associated with the dynamic balance between proinflammatory and antiinflammatory immune responses. In the early stage of infection, the proinflammatory cytokines TNF-α and IFN-γ increase rapidly, which was critical for the host to control parasitemia [69]. However, excessive proinflammatory responses may become a driving factor of immunopathological damage. Meanwhile, the antiinflammatory cytokine IL-10 played a key protective role in limiting the development of severe malaria by suppressing excessive inflammatory responses [70]. Therefore, by detecting the serum levels of TNF-α, IFN-γ, and IL-10, this study aimed to investigate the effects of DSF and TTM treatment on systemic inflammatory responses in malaria-infection-induced liver injury. Our results showed that DSF treatment significantly increased the levels of TNF-α and IFN-γ while decreasing IL-10 levels in *Pb*-infected mice. In contrast, TTM treatment significantly decreased TNF-α and IFN-γ levels and increased IL-10 levels. These findings suggest that DSF and TTM may regulate the proinflammatory/antiinflammatory balance in opposite directions, thereby influencing the severity of malaria infection-induced liver injury. This was consistent with the our previous report [17], further supporting the notion that DSF and TTM may affect immune balance in malaria infection through regulating copper homeostasis. Collectively, our data indicate that cuproptosis-driven HSC activation exacerbates hepatocyte apoptosis, likely through a paracrine mechanism mediated by HSC-derived proinflammatory factors.

From an evolutionary perspective, the observation that *Plasmodium* infection induced hepatic copper deposition raised the question of whether this reflects active parasite manipulation or a passive byproduct of heme degradation. Multiple lines of evidence indicated that *Plasmodium* actively regulated host copper homeostasis rather than passively accumulating copper as a byproduct of heme metabolism. First, the *Plasmodium* parasite possessed a complete and functional copper transport system, including parasite-encoded copper transporters (e.g., CuP-ATPase) and metallochaperones (e.g., *Pf*Cox17), demonstrating that *Plasmodium* can actively import and handle copper [71, 72]. Second, copper was essential for parasite development: copper chelation arrested intraerythrocytic growth of *P. falciparum*, confirming a critical metabolic requirement [19]. Moreover, it was shown that hypnozoites express heavy metal transporters and are killed by the copper chelator neocuproine [73], while disruption of a parasite copper transporter had no effect on blood-stage growth but abolishes gametocyte fertility, thereby blocking mosquito transmission [74]. Such stage-specific requirements were difficult to reconcile with a passive byproduct of heme degradation, which primarily involved iron and follows independent pathways [75]. If copper deposition were simply a consequence of hemoglobin digestion, one would expect copper changes to parallel iron changes and lack stage-specific regulation—contrary to what is observed. Together, these findings suggested that *Plasmodium* actively acquire and utilize host copper for critical life cycle transitions, particularly in the liver and transmission stages. The liver, as the body’s primary copper storage organ, naturally became a target of this regulation. This interpretation aligned with the broader concept of nutritional immunity, where the net hepatic copper accumulation reflected a dynamic equilibrium between host restriction and parasite acquisition.

In line with the established roles of DSF as a copper ionophore that promoted cuproptosis and TTM as a copper chelator that antagonized it [76–78], our previous studies had shown that the observed effects were predominantly attributable to modulation of copper homeostasis and cuproptosis, and that this regulation mediated the severity of lung and brain tissues during malaria infection [17, 18]. In the present study, the opposing effects of DSF and TTM on disease severity, hepatic copper accumulation, and cuproptosis marker expression (Figs. 5, 6), together with the specific co-localization of cuproptosis markers in activated HSCs, provided strong evidence for copper-dependent mechanisms. Moreover, the absence of hepatotoxicity in uninfected mice confirmed that these effects depend on the pathological context of infection-induced copper overload. Having established that DSF and TTM exerted their effects primarily through copper-dependent mechanisms, it was worth noting that neither drug is absolutely selective for copper. DSF can form complexes with other divalent cations, such as Zn^2^⁺ [79–81], and TTM, while primarily a copper chelator, may theoretically interact with other metal ions under certain conditions [82, 83]. Because several hepatic matrix metalloproteinases (MMPs) that regulate extracellular matrix turnover and inflammation were Zn^2^⁺‑dependent, DSF‑mediated Zn^2^⁺ chelation could additionally suppress MMP activity (e.g., MMP‑2 and MMP‑9), thereby impairing fibrotic matrix degradation [84, 85]. In our malaria model, this effect may synergize with copper overload‑driven HSC activation to promote collagen accumulation. By contrast, TTM removed copper with high selectivity over zinc, consistent with its protective role [86]. Notably, in noninfectious fibrosis models where copper overload is absent, DSF has been reported to attenuate liver fibrosis—highlighting that the net effect of DSF is strongly context dependent and tilted toward injury when copper‑driven cuproptosis predominates [87]. Accordingly, potential interference from other divalent cations cannot be entirely excluded, and our study did not systematically measure changes in zinc, iron, or other metal ions following DSF/TTM intervention. Future studies employing copper-specific fluorescent probes, genetic approaches targeting copper transporters (CTR1/ATP7B), or comprehensive metallomic analyses will be valuable to further delineate the independent role of cuproptosis in malaria-associated liver injury.

TTM exhibited direct anti-plasmodial activity, as evidenced by reduced peripheral and hepatic parasite burden, consistent with previous reports [19], where TTM disrupted copper homeostasis in *P. falciparum*, causing irreversible growth arrested during trophozoite-to-schizont transition. However, this alone did not fully account for the observed hepatoprotection. Critically, DSF and TTM—both of which possess reported anti-plasmodial activity [19, 88, 89]—exerted opposite effects on liver injury: DSF exacerbated pathology, whereas TTM ameliorated it. This dichotomy indicated that host copper homeostasis independently regulated liver damage, independent of parasite load. In support of this, our in vitro data showed that TTM directly downregulated cuproptosis and fibrosis markers in HSCs in the absence of immune cells. Moreover, published evidence demonstrated that copper ions directly promoted HSC proliferation and activation via oxidative stress and ASIC1a-mediated ER stress [90, 91], and that copper overload or cuproptosis directly induced HSC activation and severe liver fibrosis in animal models [92, 93]. Collectively, these findings suggest that TTM reduces liver injury through synergistic mechanisms: direct anti-parasitic activity, direct inhibition of HSC cuproptosis, and modulation of systemic immune responses toward an antiinflammatory profile.

Together, our findings establish copper-overload-induced cuproptosis as a key driver of HSC activation and acute liver injury in murine malaria. However, it is important to consider that these results were obtained using a blood-stage inoculation model, whereas natural infection begins in the liver—a distinction with potential implications for therapeutic translation. First, liver-stage parasites are highly susceptible to metal chelation. It was demonstrated that in *Plasmodium cynomolgi* liver stages, hypnozoites express heavy metal transporters, and the copper chelator neocuproine was found to be cidal against all liver-stage parasites [73]. This finding strongly suggests that copper chelation initiated during the liver stage could theoretically interrupt infection before it establishes in the bloodstream, thereby exerting a prophylactic effect. Second, the liver is the primary organ for copper metabolism, with hepatocytes highly expressing copper transporters (ATP7B, CTR1) and exhibiting active copper metabolism [32, 94]. This implies that copper chelators administered during the liver stage would achieve higher local concentrations in the liver parenchyma, potentially more effectively blocking copper-dependent processes required for parasite development. In contrast, mature erythrocytes lack nuclei and have limited metabolic activity [95], providing a less favorable environment for chelator accumulation. Third, the massive replicative capacity of liver-stage parasites offers a critical intervention window. Upon successful invasion, sporozoites undergo extensive replication within hepatocytes, generating tens of thousands of merozoites that are subsequently released into the bloodstream [96]. Effective blockade during this amplification step could contain infection before widespread dissemination, potentially yielding greater efficacy than interventions initiated after parasites have already amplified exponentially in the blood. This principle has been successfully demonstrated with other liver-stage active compounds. Fourth, while blood-stage parasites also require copper for development [36, 71, 97], whether liver-stage parasites have heightened copper dependency due to rapid proliferation and mitochondrial biogenesis remains an open question warranting future investigation. Additionally, the interplay between liver and blood stages in natural infection—where existing blood-stage parasitemia can impair subsequent liver-stage development through hepcidin-mediated mechanisms—adds complexity that our acute model does not capture. Despite these considerations, the blood-stage model remains highly relevant for understanding therapeutic intervention in symptomatic malaria, as patients typically seek medical attention during the erythrocytic stage when parasitemia and pathology are manifest. The intraperitoneal inoculation of parasitized erythrocytes offers a technically simple, dose-controlled, and highly reproducible methodology extensively validated in hepatic pathology research.

Although our pharmacological interventions—using the copper ionophore DSF and copper chelator TTM—definitively identified cuproptosis as a critical determinant of malaria-associated acute liver injury severity, several key mechanistic questions remain unresolved. First, the spatiotemporal dynamics of copper accumulation within HSCs during infection await direct quantification using advanced imaging or analytical techniques beyond the scope of this study. Second, the precise molecular cascade linking hepatic copper overload to cuproptosis induction—including specific copper-binding partners, FDX1 activation kinetics, and downstream signaling events that drive HSC activation and fibrogenesis—had not been fully elucidated. Third, although this study established a causal role of cuproptosis through biochemical and pharmacological approaches, we did not employ advanced fluorescent copper sensors to directly visualize Cu^2^⁺ dynamics at the cellular level [98, 99] due to current limitations in instrumentation and funding. Such techniques offer the advantage of oxidation-state-specific detection and real-time monitoring of labile copper pools in live cells. Future studies incorporating these tools will help to further dissect the spatiotemporal heterogeneity of copper homeostasis in specific hepatic cell populations during malaria infection and to distinguish between Cu(I) and Cu(II) contributions to cuproptosis induction. Fourth, to further strengthen the mechanistic link between copper dysregulation and liver injury, alternative approaches should be considered. Dietary copper restriction had been shown to modulate hepatic pathology in rodent fibrosis models and could provide orthogonal validation of our findings [100]. More elegantly, generating transgenic *P. berghei* lines with impaired copper transport—for instance, disrupting the copper-transporting P-type ATPase (CuTP) known to be critical for parasite fertility and copper efflux [71, 74]—would allow for dissection of parasite-intrinsic versus host-derived contributions to copper overload. Such genetic approaches could definitively establish whether parasite-driven copper dysregulation is required for inducing cuproptosis and HSC activation. While these strategies were beyond the scope and funding capacity of the current study, they will be prioritized in future investigations to provide a more comprehensive understanding of copper’s role in malaria-associated liver fibrosis. Addressing these knowledge gaps will be essential to comprehensively understand cuproptosis as a central pathogenic mechanism in malaria-induced liver injury and to inform the rational development of targeted therapies that modulate copper homeostasis or cuproptosis-related signaling.

## Conclusions

This study identified copper-overload-induced cuproptosis as a pivotal pathogenic mechanism that exacerbated acute liver injury in murine malaria. We provided direct evidence of hepatic copper accumulation and activation of the cuproptosis pathway in infected mice, accompanied by a marked expansion of activated HSCs co-expressing key cuproptosis markers. Importantly, pharmacological intervention established a clear causal relationship: the copper ionophore DSF enhanced cuproptosis, promoted HSC activation and fibrosis, and worsened liver injury, whereas the copper chelator TTM suppressed cuproptosis, inhibited HSC activation, and alleviated hepatic pathology. Although the detailed molecular events connecting cuproptosis to HSC activation require further investigation, our findings may highlight the therapeutic potential of targeting the cuproptosis–HSC activation axis to ameliorate parasite-induced acute liver injury.

## Supplementary Information

Supplementary material 1. Table 1. Primer sequences used in the qPCR analysis of target cytokines. All forward (F) and reverse (R) primers for qPCR analysis were designed and synthesized by Sangon Biotech (Shanghai, China).

## Data Availability

All datasets generated for this study are included in the manuscript and additional file.
