# Supplementary material for: Copper overload drives hepatic stellate cell activation via cuproptosis to exacerbate acute liver injury in murine malaria
Source: Parasit Vectors. 2026 Jun 8;19:331. doi: 10.1186/s13071-026-07476-0 (PMC13463495; doi:10.1186/s13071-026-07476-0)
Supplement: Supplementary file 1 [file 13071_2026_7476_MOESM1_ESM.docx]

| Genes | Primers ^a^ |
| --- | --- |
| α-SMA | F: 5’-TCAGGGAGTAATGGTTGGAATGGG-3’  R: 5’-GGTGATGATGCCGTGTTCTATCG-3’ |
| COL1A1 | F: 5’-AGGCGAACAAGGTGACAGAGG-3’  R: 5’-GGAGAACCAGGAGAACCAGGAG-3’ |
| TGF-β | F: 5’-GGAAGAATACACCACCAGCAGTC-3’  R: 5’-CACGGTAGCAGTAGAAGATGATGATG-3’ |
| PDGF | F: 5’-AGTGGCTACATCATCCCCCT-3’  R: 5’-CCGAAGTCTGTGAGCTGTGT-3’ |
| IFN-γ | F: 5’-TCAAGTGGCATAGATGTGGAAG-3’  R: 5’-TGCTGTTGCTGAAGAAGGTAGT-3’ |
| HGF | F: 5’-CACACCGGCACAAGTTCTTG-3’  R: 5’-AAGAGTGTAGCACCATGGCC-3’ |
| TNF-α | F: 5’-GCCTCTTCTCATTCCTGCTTGTGG-3’  R: 5’-GTGGTTTGTGAGTGTGAGGGTCTG-3’ |
| IL-1β | F: 5’-TCGCAGCAGCACATCAACAAG-3’  R: 5’-TCCACGGGAAAGACACAGGTAG-3’ |
| FDX1 | F: 5’-CGGAGCGGGAACTGCCATC-3’  R: 5’-TCTCGCCATCTCGGTTCTTGAAG-3’ |
| DLAT | F: 5’-CTCAGGCATGTATTCTGGCAATCG-3’  R: 5’-TCCAACTGCTCCATCCACAACTC-3’ |
| DLST  *Pb*A 18S rRNA | F: 5’-GGGTCTCGTGGTTCCTGTCATC-3’  R: 5’-TCCGTTGCTGATGGTGAAGGTAC-3’  F: 5’-ACGGGGAGCAAGAGCAGTATTTC-3’  R: 5’-CCCCACCAAAACCTGCCTTTATTG-3’ |
| GAPDH | F: 5’-ACGGCAAATTCAACGGCACAG-3’  R: 5’-ACACCAGTAGACTCCACGACATAC-3’ |

**Supplementary Table 1.** **Primer sequences used in the qPCR analysis of target cytokines.**

Notes: ^a^ F, Forward primer; R, Reverse primer; Primers were custom-synthesized by Sangon Biotech (Shanghai, China).
